# Phosphate solubilizing *Pseudomonas* and *Bacillus* combined with rock phosphates promoting tomato growth and reducing bacterial canker disease

**DOI:** 10.3389/fmicb.2024.1289466

**Published:** 2024-05-03

**Authors:** Mohamed Bakki, Badra Banane, Omaima Marhane, Qassim Esmaeel, Abdelhakim Hatimi, Essaid Ait Barka, Khalid Azim, Brahim Bouizgarne

**Affiliations:** ^1^Laboratory of Plant Biotechnology “Biotechnologies Végétales”, Faculty of Sciences, University Ibn Zohr (UIZ), Agadir, Morocco; ^2^Unité de Recherche Résistance Induite et Bio Protection des Plantes, EA 4707 – USC INRAe1488, UFR Sciences Exactes et Naturelles, Moulin de la Housse, University of Reims Champagne-Ardenne, Reims, France; ^3^Integrated Crop Production Research Unit, Regional Center of Agricultural Research of Agadir, National Institute of Agricultural Research, Rabat, Morocco

**Keywords:** *Bacillus*, biofertilization, rock phosphate, plant growth promotion, *Pseudomonas*, solubilization, induced systemic resistance

## Abstract

Nowadays, sustainable agriculture approaches are based on the use of biofertilizers and biopesticides. Tomato (*Solanum lycopersicum* L.) rhizosphere could provide rhizobacteria with biofertilizing and biopesticide properties. In this study, bacteria from the rhizosphere of tomato were evaluated *in vitro* for plant growth promotion (PGP) properties. Five *Pseudomonas* isolates (PsT-04c, PsT-94s, PsT-116, PsT-124, and PsT-130) and one *Bacillus* isolate (BaT-68s), with the highest ability to solubilize tricalcium phosphate (TCP) were selected for further molecular identification and characterization. Isolates showed phosphate solubilization up to 195.42 μg mL^−1^. All isolates showed phosphate solubilization by organic acid production. The six isolates improved seed germination and showed effective root colonization when tomato seeds were coated with isolates at 10^6^ cfu g^−1^ in axenic soil conditions. Furthermore, the selected isolates were tested for beneficial effects on tomato growth and nutrient status in greenhouse experiments with natural rock phosphate (RP). The results showed that inoculated tomato plants in the presence of RP have a higher shoot and root lengths and weights compared with the control. After 60 days, significant increases in plant Ca, Na, P, protein, and sugar contents were also observed in inoculated seedlings. In addition, inoculated tomato seedlings showed an increase in foliar chlorophyll a and b and total chlorophyll, while no significant changes were observed in chlorophyll fluorescence. In greenhouse, two *Pseudomonas* isolates, PsT-04c and PsT-130, showed ability to trigger induced systemic resistance in inoculated tomato seedlings when subsequently challenged by *Clavibacter michiganensis* subsp. *michiganensis*, the causal agent of tomato bacterial canker. High protection rate (75%) was concomitant to an increase in the resistance indicators: total soluble phenolic compounds, phenylalanine-ammonia lyase, and H_2_O_2_. The results strongly demonstrated the effectiveness of phosphate-solubilizing bacteria adapted to rhizosphere as biofertilizers for tomato crops and biopesticides by inducing systemic resistance to the causal agent of tomato bacterial canker disease.

## 1 Introduction

The exponentially growing world population, which is estimated to reach 9.5 billion by 2050, will imply more food needs and more demand of agricultural productivity, particularly vegetable crops. Tomato (*Solanum lycopersicum* L.) is one of the most consumed and economically important vegetable crops in the world with a world production of over 186 million tons (FAOSTAT 2022). Plant nutrition is one of the major concerns for modern agriculture. Nitrogen (N), phosphorus (P), and potassium (K) are major growth-limiting macronutrients for plant growth. Sustainable agricultural practices should be encouraged, and development of new biological tools is needed. Application of beneficial soil microbia, either alone or combined with natural nutrient sources such as rock phosphate, represent a promising strategy. On the other hand, tomato is attacked by the gram-positive bacterium *Clavibacter michiganensis* subsp. *michiganensis* (*Cmm*), which causes bacterial canker on tomato stem, leaves and fruits. The disease is considered the major threat for tomato cultivation all over the world, as it can cause economic losses that can reach up to 100% of production (Chang et al., 1992; Wang et al., 2022). Nowadays, two main strategies are commonly adopted for enhancement of plant growth and disease management: (i) development of new cultivars with enhanced growth and nutritional qualities and resistance to phytopathogens (ii) use of chemical fertilizers and pesticides. However, for tomato bacterial canker, no resistant cultivars are available, and no chemical treatments are efficient to combat this disease.

Phosphorus is the second most important nutrient for the plant growth after nitrogen, which plays a crucial role in energy transfer, photosynthesis, signal transduction, and respiration (Ham et al., 2018; Bouizgarne, 2022). It accounts for between 0.2 and 0.8% of the dry weight of plants, and adequate availability of this nutrient is required in many physiological and biochemical plant activities. Phosphorus exists in two forms in the soil, organic and inorganic phosphates. Insoluble forms of iron, aluminum, and calcium as aluminum phosphate (Al_3_PO_4_) and ferrous phosphate (Fe_3_PO_4_) exist in acidic soils and as calcium phosphate (Ca_3_PO_4_)_2_ in calcareous soils. Natural rock phosphate (RP) is composed of calcium hydroxyapatite [Ca_10_ (PO_4_)_6_ OH], with only small fractions of phosphate anions, H_2_PO_4_^−^, HPO_4_^2−^, and PO_4_^3−^. In addition, soluble forms are rapidly converted into insoluble complex forms with iron, aluminum, or calcium. All these mechanisms render phosphorus into an insoluble form which is not directly available for plant uptake (Adnan et al., 2020). This conversion is also responsible for the loss of approximately 75% of chemical P fertilizer, added to soils each year, which rapidly becomes unavailable for plants. Consequently, although abundant in natural soils, P-availability in the soil solution is often insufficient as most soils are poor in phosphorus, which is considered a limiting factor for plant nutrition (Bouizgarne, 2022). Its deficiency severely restricts plant growth and yields. Under a limited P-availability, plant root system becomes concentrated in the topsoil, resulting in inhibited elongation of primary root and stimulation of lateral root initiation. In the other hand, direct application of rock phosphate as fertilizer is not effective in most soils as plants could not extract phosphorus from these forms, making increasing phosphorus use efficiency a major challenge in intensive agricultural production systems.

This issue could be addressed efficiently by using soil microorganisms, particularly those living in the vicinity of the roots, also called plant growth-promoting rhizobacteria (PGPR) in an eco-environmental context. PGPR have attracted more attention during last decades as they have the potential to contribute to acceptable crop yields while preserving soil health. They are crucial in maintaining agricultural soil function because they contribute to soil processes such as stabilizing soil aggregates, improving soil structure, breakdown of organic matter, and cycling of nutrients such as carbon, nitrogen, phosphorus, and sulfur. In addition, they act by providing growth compounds to plants, helping plants to uptake soil nutrients, or preventing them from being infected by phytopathogens (Bouizgarne, 2013; Riaz et al., 2021; Zhang et al., 2021).

Among PGPR, bacteria that are able to solubilize insoluble phosphate forms, named phosphate solubilizing bacteria (PSB), dwell in most soils and potentially represent 40% of the culturable population (Richardson, 2001). PSB have been considered important for making soluble phosphorus forms available to plants by solubilizing insoluble inorganic phosphates or mineralizing organic phosphate compounds into soluble forms (Gyaneshwar et al., 2002; Rodríguez et al., 2006). They also prevent the released P from being immobilized again (Richardson et al., 2011). The most known mechanism by which PSB act is by generating organic acids that are able to solubilize complex mineral phosphates, including calcium phosphates in alkaline soils and RP (Alori et al., 2017; Bargaz et al., 2021). The mechanisms of PGPR-mediated enhancement of plant growth and yield of many crops are not yet fully understood. However, some PSB might actively contribute to the plant health by other mechanisms such as producing phytohormones or active substances against some specific plant pathogens or reducing ethylene synthesis (Bouizgarne, 2013). Bacterial strains belonging to genera *Bacillus, Pseudomonas*, and *Serratia* were reported for their ability to solubilize insoluble mineral phosphate compounds, such as dicalcium and tricalcium phosphate and rock phosphate (Ahmad et al., 2022; Bouizgarne et al., 2023). In particular, bacteria belonging to *Pseudomonas* species have frequently been isolated from the plant rhizosphere, and several have been reported as plant growth-promoting rhizobacteria (PGPR), considering their ability to increase P-availability in plants, thus helping to sustain crop health and productivity (Bouizgarne, 2013). In addition, the opportunity of improving the P uptake of crops by artificial inoculation with P-solubilizing rhizobacteria has been shown as an attractive plan for research (Azaroual et al., 2020).

The demand for chemical-free food is rising among consumers and sparked the research for environmentally safe techniques in order to reduce the reliance on chemicals. It is therefore recommended to search for alternative methods such as using phosphate solubilizing PGPR adapted to the rhizosphere of plant roots combined with natural rock phosphate in order to enhance the effectiveness of rock phosphate as fertilizer. The application of phosphate solubilizing bacterial strains from Moroccan rock phosphate, for tomato growth promotion has been reported (Bouizgarne et al., 2023). This is the first report of the use of *Pseudomonas* or *Bacillus*, combined with rock phosphate, for tomato growth promotion and protection against the causal agent of bacterial canker, *Clavibacter michiganensis* subsp. *michiganensis,* by improving host resistance mechanisms.

Thus, the present study aims (i) to screen for efficient phosphate, potash, and zinc solubilizing bacteria from tomato rhizosphere soil in addition to indole acetic acid (IAA) production, (ii) investigate the beneficial effects of their combination with natural rock phosphate (RP) on tomato growth and nutrient uptake in greenhouse experiments, and (iii) biocontrol of tomato bacterial canker caused by *Cmm* through triggering induced systemic resistance in inoculated tomato seedlings.

## 2 Materials and methods

### 2.1 Isolation of bacteria and screening for P, K, and Zn solubilizing activity

#### 2.1.1 Screening for phosphate solubilizing bacteria

Bacterial strains were isolated from two soil areas in the Souss-Massa region, Morocco. Soil samples were collected from the root system of five healthy tomato plants. Soil dilutions were performed, and 0.1 mL was spread on the King B medium (per liter: proteose peptone 20 g; K_2_HPO_4_ 1.5 g; MgSO_4_.7H_2_O 1.5 g; pH 7.2). After incubation at 28°C for 48 h, bacteria were stained with the Gram staining method and checked for cytochrome-C oxidase (oxidase test). Bacteria showing fluorescence under UV at 365 nm were considered as fluorescent pseudomonads. The capacity of isolates to solubilize inorganic phosphate was tested on National Botanical Research Institute’s phosphate (NBRIP) growth medium [per liter: 10 g glucose, 0.1 g (NH_4_)_2_SO_4_, 0.2 g KCl, 0.25 g MgSO_4_, 7H_2_O, 5 g MgCl_2_, 6H_2_O, and 5 g tri-calcium phosphate (TCP)] (Nautiyal, 1999). Appearance of halos around colonies reflected tri-calcium phosphate solubilization. The experiment was performed in four replicates. Halo diameter around colony and the colony diameter were measured, and the solubilization index (PSI) was calculated according to the following formula:

$$PSI=\left[\begin{array}{l} \mathrm{Diameter}\;\mathrm{of}\;\mathrm{solubilization}\;\mathrm{halo}+\\ \mathrm{Colony}\;\mathrm{diameter} \end{array}\right]/\mathrm{Colony}\;\mathrm{diameter}$$

Quantitative estimation of phosphate solubilization was performed in liquid NBRIP medium; 20 mL of NBRIP containing TCP was inoculated with 100 μL of bacterial suspension from a 24-h preculture in King B medium (KB) at a final concentration of 10^6^ cfu mL^−1^. The flasks were incubated at 28°C in the dark under shaking at 180 rpm in an incubator shaker (KS 3000 IC, IKA® Werke Staufen, Germany). After 48 h, phosphate solubilization was monitored by colorimetry in the culture supernatant at 600 nm, according to the Fiske and Subbarow (1925) method. A calibration curve was plotted using KH_2_PO_4_, and the pH variation was estimated by recording pH value at starting conditions and after 48 h of culture. The experiment was performed in six replicates, and the amount of P in the medium was expressed as mg L^−1^.

#### 2.1.2 Identification of organic acid by HPLC

High-performance liquid chromatography (HPLC) (Ultimate 3000 Dionex), coupled with mass spectrometry (Exactive Plus de Thermo Scientific) and equipped with a BDS Hypersil C18 (150 × 4.6 mm × 5 μm) HPLC Column, was used to identify and quantify organic acids. Flasks containing 20 mL of liquid NBRIP with TCP as P source were inoculated with 100 μL of standardized bacterial suspension at a final concentration of 10^6^ cfu mL^−1^ and incubated at 28°C in the dark under shaking at 180 rpm. After 48 h, samples were collected, filtered through a 0.22 μm cellulose membrane, and injected into the chromatographic column. Detection of organic acids was performed using the following organic acids (Supelco/Sigma–Aldrich) as analytical standards with typical retention time means as follows: 2-ketogluconic acid (2.10 min), phytic acid (3.25 min), gluconic acid (3.75 min), tartaric acid (3.93 min), quinic acid (4.03 min), oxalic acid (4.25 min), succinic acid (4.85 min), DL-iso-citric acid (4.86 min), ascorbic acid (5.05 min), maleic acid (5.56 min), citric acid (7.51 min), fumaric acid (8.10 min), shikimic acid (8.71 min), propionic acid (9.00 min), malonic acid (9.27 min), and benzoic acid (12.5 min). The mobile phase was 0.1% H_3_PO_4_ (pH 1.81) with a flow rate of 0.5 mL min^−1^ and a 100 μL injection per sample, according to Marra et al. (2015). The acquisition time of the chromatograms was estimated to be 30 min with 30 min of intervals between runs. Detection was performed by UV at 210 nm with a diode array detector (DAD).

#### 2.1.3 Screening for potassium solubilization

Potassium solubilization was carried out by bacterial isolates on Aleksandrow agar medium (Rajawat et al., 2014) containing 5.0 g/L glucose, 0.5 g/L MgSO_4_, 7H_2_O, 0.1 g/L CaCO_3_, 0.006 g/L FeCl_3_, 2.0 g/L Na_2_ HPO_4_, and feldspar (2.0 g/L) as sole K source. The plates were then incubated for 2 days at 28°C. Appearance of yellow halos around colonies reflected potassium solubilization. The experiment was performed in four replicates. Halo diameter around each colony and the colony diameter were measured. Potassium mineral solubilizing index (KSI) was calculated as follows:

$$KSI=\left[\begin{array}{l} \mathrm{Diameter}\;\mathrm{of}\;\mathrm{solubilization}\;\mathrm{halo}+\\ \mathrm{Colony}\;\mathrm{diameter} \end{array}\right]/\mathrm{Colony}\;\mathrm{diameter}$$

#### 2.1.4 Screening for zinc solubilization

The zinc solubilization was investigated in Bunt and Rovira medium (Bunt and Rovira, 1955) (glucose: 20.0 g, peptone: 1.0 g; yeast extract: 1.0 g, (NH_4_)_2_ SO_4_: 0.50 g; K_2_ HPO_4:_ 0.40 g; MgCl_2_: 0.10 g, FeCl_3_: 0.01 g, distilled water 1.000 mL, and adjusted pH 6.7) containing 0.1% insoluble zinc oxide. Fresh cultures of bacteria were spot inoculated at the center of the plate in triplicates with toothpicks and incubated for 72 h in the dark at 26°C. The experiment was performed in four replicates. The appearance of halo zones around colonies reflected zinc solubilization. Halo diameter around each colony and the colony diameter were measured. The zinc solubilizing index (ZSI) was calculated as follows:

$$ZSI=\left[\begin{array}{l} \mathrm{Diameter}\;\mathrm{of}\;\mathrm{solubilization}\;\mathrm{halo}+\\ \mathrm{Colony}\;\mathrm{diameter} \end{array}\right]/\mathrm{Colony}\;\mathrm{diameter}$$

### 2.2 Screening for indole-3-acetic-acid production

Production of IAA was demonstrated according to Bric et al. (1991). Isolates were cultivated in Luria–Bertani (LB) medium (per liter: tryptone 10 g; yeast extract 5 g; NaCl 5 g; pH 7.2) and incubated at 28°C for 2 days, and then, positive colonies were revealed by Salkowski reagent. For quantitative measurement, liquid LB medium containing 0.1 g/L of L-Trp was seeded with isolates and incubated for 3 days at 28°C. Amount of IAA was determined according to Gravel et al. (2007). A calibration curve was obtained by synthetic IAA (Sigma–Aldrich). The experiment was performed in six replicates, and amounts of IAA were expressed in μg mL^−1^.

### 2.3 Effect of bacterial isolates on tomato seed germination

Bacterial isolates were cultivated for 48 h days in King B broth and incubated at 28°C on a rotating shaker at 180 rpm in an incubator shaker (KS 3000 IC, IKA® Werke Staufen/Germany). Tomato seeds (cultivar calvi) were surface sterilized using 2% sodium hypochlorite and subsequently washed with distilled water. Then, 50 tomato seeds were placed in sterile Petri dishes containing wet sterile paper filter. Seeds were coated with 1 mL of a mixture containing culture suspension from each of the six bacteria at a concentration of 10^6^ cfu mL^−1^, 0.5% carboxymethyl cellulose (CMC), and 0.1% RP. The composition of RP was evaluated by using scanning electron microscopy (SEM–EDX, JSM IT-100, JEOL, USA) as follows: C: 7.1%, O: 48.6%, F: 11.0%, Na: 1.8%, Al: 1.7%; Si: 3.5%, P: 8.9%, and Ca: 17.1%. RP contains particles with an average size of 377 μm. pH of H_2_O (1.1) was 6.86 and electrical conductivity was 488 mmho cm^−1^. It contains approximately 5% total phosphorus and low amount of assimilable phosphorus (P-Olsen) (P_2_O_5_ < 18 mg/Kg). It contains low amount of organic carbon (0.23%) and organic matter (0.40%) and 99.5% of mineral matter with 5.72% total limestone. The C/N ratio was 3.55. RP contains low amount of nitrogen (0.012%), calcium (CaO) 1.93 g/kg, and potassium (0.01 g/kg). RP magnesium (MgO) content was 0.34 g/kg. The mixture was added to seeds under shaking condition until a fine coating appeared on seeds. The plates were placed in a germination chamber at 28 ± 2°C for 12 days. Germinated seeds of negative control were coated with bacteria-free CMC. The percent of germinated seeds was determined after 15 days (International Seed Testing Association, 2009). The experiment was performed with three replicates.

### 2.4 Greenhouse experiments

#### 2.4.1 Rhizosphere colonization by selected bacteria

To investigate the ability of the selected isolates to colonize the roots of tomato plants, six tomato seeds (cv. calvi) were first surface disinfected with 2.5% sodium hypochlorite solution for 3 min and then coated with 1 mL of a mixture containing culture suspension from each of six bacteria at a concentration of 10^6^ cfu mL^−1^, 0.5% carboxymethyl cellulose (CMC), and 0.1% RP and germinated in Petri dishes. After germination, seedlings were transferred in a mixture of soil and peat (2,1 w,w) and cultured in axenic conditions. Soil characteristics were as follows: silt (6.70%), clay (4.15%), sand (87.32%), pH (H_2_O): 8.57, and electrical conductivity (EC) at 25°C (0.28 mS cm^−1^). Water retention capacity was 30.71%, and total organic carbon was 0.4%. Rhizosphere colonization was determined after 15 days by uprooting seedlings and removing root adhering soil by washing three times. The homogenate was then stirred in 9 mL sterile water for 5 min. Then, serial decimal dilutions were carried out in sterile physiological water. Overall, 0.1 mL of each dilution was spread on King B agar medium and incubated at 28°C for 24 h, and the growth of the bacterial isolates was estimated by counting colonies. Rhizosphere colonization was expressed as colony forming unit (cfu) g^−1^ soil. Each experiment was performed in triplicate.

#### 2.4.2 Effects on plant growth parameters

In addition, after germination, seedlings were transferred to disinfected pots (one seedling per pot) containing 5 kg of a mixture of soil and peat (2,1 w,w) amended with RP at 1 g/kg mixture. Plants were placed in an experimental greenhouse where the average temperature was 35°C during the day and 10–18°C at night, a photoperiod of 16 h day/8 h night, and an average relative humidity of 67.5%. Seedlings were watered daily with sterilized water. To check for the ability of bacteria to promote the growth in tomato, the following experiments were carried out: (i) control seedlings: tomato seedlings were grown in the mixture of soil and peat and amended with RP (1 g/kg) as a source of phosphorus and (ii) bacterized seedlings: tomato seedlings were grown in the mixture of soil and peat and amended with RP (1 g/kg) and inoculated at plant rhizosphere with 20 mL of bacterial suspension from each selected isolate at 10^6^ mL^−1^. Each month, the treatment with the bacteria at plant rhizosphere was renewed. Thirty seedlings were used for each experiment in three replicates. Tomato plants were harvested after 60 days, and then, the length of the seedling shoots and roots and the shoot and root fresh and dry weight were measured. In addition, the following physiological parameters were measured:

#### 2.4.3 Effects on chlorophyll content and chlorophyll fluorescence

The effect of the different treatments on total chlorophyll content was measured in youngest fully expanded leaves, which were detached from tomato plants immediately after harvesting. Measurements were performed according to Arnon (1949). Four repetitions were performed per treatment, and chlorophyll contents were expressed in mg g^−1^ DM. The chlorophyll fluorescence (Fv/Fm) (Maxwell and Johnson, 2000) was measured using a portable fluorometer (Handy PEA fluorimeter, Hansatech instruments, Norfolk, UK). This parameter was measured on well-developed leaves of the same rank (seven repetitions per treatment). Clamps were placed on the upper side of the leaves for 20 min, to keep the leaves in the dark before measurement.

#### 2.4.4 Effects on proteins and total soluble sugars

The extract was prepared by mixing 100 mg of the aerial part (stem+ leaves) of each plant sample with 4 mL of 0.1 M phosphate buffer pH 6 containing 5% insoluble polyvinylpyrrolidone and 0.1 mM ethylenediaminetetraacetic acid (EDTA). The protein content in the supernatant was measured according to Bradford (1976). Total soluble sugar was determined according to Dubois et al. (1956). The results were expressed in mg g^−1^ of fresh matter.

#### 2.4.5 Effects on mineral elements contents

Sodium (Na^+^), potassium (K^+^), calcium (Ca^2+^), and phosphorus (P) were measured after mineralization of the plant shoot and root material. Sodium (Na^+^), potassium (K^+^), and calcium (Ca^2+^) cations were measured by flame photometer PFP7 (Jenway, United Kingdom), according to Tuna et al. (2007). To measure phosphorus in leaves, the phospho-molybdate blue method was used (Olsen and Sommers, 1982). In brief, an aliquot of the solution (1 mL) was added to 5 mL of the following reagent mixture: ammonium molybdate (2.5%), hydrazine sulfate (0.15%), and 4 mL of distilled water. After heating in a water bath at 70°C for 10 min and cooling for 5 min, optical density of the samples was measured at λ = 820 nm with a UV/VIS spectrophotometer (UV-3100PC, VWR®, United States).

### 2.5 Biocontrol of tomato bacterial canker by induced systemic resistance

#### 2.5.1 Ability of bacteria to grow inside root tissues and on root exudates

Tomato seedlings were used to test the growth of six bacterial isolates, PsT-04c, PsT-94s, BaT-86s, PsT-116, PsT-124, and PsT-130, on root tissues and crude root exudates. Tomato seedlings (15 days old) were removed carefully from pots containing the mixture of soil and peat and washed three times with sterilized water. The roots were carefully cleaned with cotton soaked in alcohol, surface disinfected with 0.4% sodium hypochlorite, and rinsed extensively with sterile deionized water. 10 μL of each bacterial suspension at a concentration of 10^6^ bacteria mL^−1^ was injected into the main roots of six tomato seedlings by using a Hamilton micro syringe. Seedlings were then transferred to aseptic flasks containing 450 mL of sterile distilled water, inoculated with 10^6^ mL^−1^ of each of the six bacteria, and hydroponically grown in a growth chamber at 25 ± 2°C in natural light with 16-h photoperiod for 15 days. The control consisted of six seedlings injected only with sterile water and transferred to uninoculated flasks under the same conditions. To check for the ability of isolates to grow in root tissues, after 15 days, roots were ground in a mortar at 4°C; 1 g of the homogenate was stirred in 9 mL of sterile water for 5 min. Then, serial decimal dilutions were carried out in sterile physiological water. In total, 0.1 mL of each dilution was spread on nutrient agar plates and amended with cycloheximide (40 μg mL^−1^) to inhibit the fungal growth. Three replicates were carried out by dilution. The agar plates were then incubated at 28°C for 48 h. Colonies of bacteria grown from root extracts were identified by their phenotypic characteristics, and representative colonies were identified by *16S rRNA* sequencing. Colonies were counted, and their growth was estimated as colony forming units (cfu) per gram roots.

For growth on root exudates, exudates from control seedlings were extracted and sterilized by filtration using a 0.22 μm Millipore filter. In total, 20 mL exudates were added to 80 mL sterilized distilled water and 1.5 g agar in order to prepare a solid medium (exudates agar). For each isolate, a serial dilution of non-sterilized exudate was performed, and 0.1 mL of each dilution was spread on the exudate agar, amended with cycloheximide, and incubated for 48 h at 28°C. Control consisted of root exudate-free medium. Three replicates were performed for each isolate. Colonies of bacteria grown on root exudates were counted, roots were weighed, and growth was estimated as colony forming units (cfu) per gram roots. No growth was recorded in the control without root exudates.

#### 2.5.2 Ability of bacteria to protect tomato against *Cmm* and induce systemic resistance

##### 2.5.2.1 Bacterial strains: *Pseudomonas* strains and the pathogen *Cmm*

A virulent strain of *Clavibacter michiganensis* subsp. *michiganensis* (*Cmm*) and the two selected *Pseudomonas* isolates, PsT-04c and PsT-130, were used for biocontrol experiments. *Cmm* colonies were cultivated on a slightly modified BCT medium (Ftayeh et al., 2011) containing per liter of deionized water: 2.5 g mannitol, 2.0 g yeast extract, 1.0 g K_2_HPO_4_, 0.1 g KH_2_PO_4_, 0.05 g NaCl, 0.1 g MgSO_4_ . 7H_2_O, 0.015 g MnSO_4_ . H_2_O, 0.015 g FeSO_4_ . 7H_2_O, 0.6 g H_3_BO_3_ (pH 7.1), and 15 g agar. After autoclaving and cooling at 50°C under stirring, nalidixic acid (20 mg/L), trimethoprim (100 mg/L), and 50 μL of a solution of penconazole (80 g/L) were added to the medium. To confirm the pathogenicity of the *Cmm* isolate, tomato seedlings were infected with a suspension of *Cmm* at 10^6^ bacteria mL^−1^ by depositing 50 μL droplets on the surface of the leaves and injecting 10 μL droplets into roots. After 15–20 days, *Cmm* was reisolated from infected seedlings, showing symptoms of bacterial canker.

##### 2.5.2.2 Protection of tomato seedlings against bacterial canker

For biocontrol experiments in greenhouse experiments, 1-month-old tomato seedlings obtained from seed germination were treated by either *Cmm* or *Pseudomonas* + *Cmm* and transplanted into the mixture containing soil and peat (2: 1). Treatments were carried out as follows:

- Negative control: seedlings for which roots were soaked only in sterilized water for 8 h and transplanted into pots.
- Positive control: seedlings infected by soaking in 10 mL of *Cmm* suspension at 10^6^ bacteria mL^−1^ for 8 h and transplanted into pots.
- Bacterial treatment: seedlings for which roots have been soaked in a 10 mL suspension of each of the two *Pseudomonas* strains PsT-04c and PsT-130 and subsequently infected by *Cmm*. Treatments were performed as follows: The first treatment was carried out with *Pseudomonas* (1^st^ immunization) by soaking seedlings in 10 mL of each *Pseudomonas* isolate at 10^6^ bacteria mL^−1^ for 8 h before transplanted into pots. After 1 week, seedlings were infected by inoculating 10 mL of *Cmm* suspension at 10^6^ bacteria mL^−1^ at root level. Finally, after 20 days, the second bacterial treatment, by inoculating 10 mL of each of the two *Pseudomonas* isolates at 10^6^ bacteria mL^−1^ at root level, was performed (2^nd^ immunization).

Each treatment consisted of 32 seedlings arranged in a complete random block. After 120 days of culture in greenhouse, symptoms were observed, and the mortality rate was calculated as follows:

$$\left[\begin{array}{l} \mathrm{Number}\;\mathrm{of}\;\mathrm{died}\;\mathrm{seedlings}\;\mathrm{in}\;\mathrm{each}\;\mathrm{treatment}/\\ \mathrm{Number}\;\mathrm{of}\;\mathrm{seedlings}\;\mathrm{in}\;\mathrm{control}\;\left(\%\right) \end{array}\right]\;x\;100$$

In addition, the following parameters were measured:

##### 2.5.2.3 Total soluble phenolic compounds

After 1 month of the second bacterization, control tomato seedlings, *Cmm*-infected seedlings, and seedlings treated with the two isolates PsT-04c and PsT-130 were used for the evaluation of the root-soluble phenolic compound contents, which is carried out according to the protocol described by Macheix et al. (1990). Roots (200 mg) were ground in an ice bath with 2 mL of 80% methanol before stirring for 15 min and centrifugation at 7,000 *g* for 3 min. The extraction operation was repeated three times, and the recovered supernatants were stored in the freezer (−20°C). The supernatants were used for quantitative determination of the total soluble phenolic compounds by the Folin–Ciocalteu reagent. The reaction mixture consisted of 50 μL of phenolic extracts diluted in 2 mL of distilled water and 0.5 mL of the Folin–Ciocalteu reagent (10%, v/v). After shaking, 0.5 mL of a Na_2_CO_3_ solution (20%) was added, and the mixture was incubated at 40°C for 30 min. For the colorimetric measurement, blank was prepared with 0.5 mL of Folin–Ciocalteu reagent (10%, v/v) and 0.5 mL of 20% Na_2_CO_3_. The results were read on a UV/VIS spectrophotometer (UV-3100PC, VWR®, United States) at 765 nm. The experiment was performed with three replicates, and total soluble phenolic contents were expressed in mg g^−1^ of fresh material by using a standard curve of caffeic acid.

##### 2.5.2.4 Phenylalanine ammonia-lyase activity

After 1 month of the second bacterization, the phenylalanine ammonia-lyase activity was determined according to Mozzetti et al. (1995). Tomato roots were ground in 100 mmol/L potassium borate buffer, pH 8.8, with 14 mmol/L 2-mercaptoethanol. The homogenate was then centrifuged at 13,000 g for 30 min, and 100 μL of the supernatant (enzymatic extract) was added to 1 mL of 100 mmol/L potassium borate buffer, pH 8.8, with 200 μL of 100 mmol/L L-phenylalanine. After incubation at 40°C for 60 min, the reaction was stopped by the addition of 250 μL of 5 N HCl, and the absorbance was determined at 290 nm. Cinnamic acid was used to perform a standard curve, and the protein content was determined according to Bradford (1976). The experiment was performed with three replicates, and L-phenylalanine was expressed as μg cinnamic acid mL^−1^ enzymatic extract.

##### 2.5.2.5 Hydrogen peroxide (H_2_O_2_) content

One month after the second bacterization, the H_2_O_2_ assay was performed according to Velikova et al. (2000). The fresh plant material (500 mg) was homogenized in 2 mL of 1 g/L solution of trichloroacetic acid (TCA). After centrifugation at 10,000 g for 15 min, 0.5 mL of the supernatant was added to the reaction mixture containing 0.5 mL of 10 mM potassium phosphate buffer (pH 7) and 1 mL of a 1 M solution of KI. The absorbance was measured at 390 nm. The experiment was performed with three replicates, and the H_2_O_2_ content was expressed in μg g^−1^ of fresh weight using a standard curve of H_2_O_2_, which was established under the same conditions.

### 2.6 Identification of bacterial isolates

The characterization of bacterial isolates was performed by observing phenotypic traits such as colony morphology, Gram staining, and fluorescence under UV light (254 nm). Furthermore, oxidase, tween, and Levan tests were assayed according to the methods described by Lelliott et al. (1966). Other traits were determined by using API 20NE strips (Biomérieux-France). All tests were performed in triplicate. Molecular identification of the six isolates was performed by using *16S rRNA* gene sequences as follows: the selected bacteria were grown on King B broth on a rotary shaker at 180 rpm in an incubator shaker KS 3000 IC (IKA® Werke Staufen/Germany) at 28°C for 48 h. After centrifugation, total DNA was extracted with the “MagPurix Bacterial DNA extraction kit” using the MagPurix extractor robot. Purified bacterial DNA was estimated by Nanodrop 8,000 (Thermo Fisher Scientific, USA). The bacterial *16S rRNA* gene was amplified by using the universal *16S rRNA* primers FD1 (5′- AGAGTTTGATCCTGGCTCAG-3′) and RP2 (5’-TACGGCTACCTTGTTACGACTT- 3′) (Weisburg et al., 1991). The PCR was carried out by using an ABI “Veriti, Applied Biosystems™” thermal cycler in a mixture containing 10 μL of PCR Master Mix (2X) (HS MyTaq DNA polymerase kit from Bioline), 1.5 μL of each primer, 130 ng of DNA, and purified water in a final volume of 25 μL, and the PCR program is as follows: 95°C, 2 min; (95°C, 30 s; 52°C, 30 s; and 72°C, 30 s) 35x; 72°C, 3 min. In total, 8 μL of PCR products was deposited on 1% agarose gel in the presence of the ladder of 1 kb molecular weight, and the gel was visualized by the “G Box” documentation system. Cleaned PCR products were used as a template for the cycle sequencing. Sequencing was performed using the 3130XL Dye Terminator Cycle Sequencing (DTCS) Quick Start kit (Applied Biosystems). The thermocycling conditions were as follows: 25 cycles of 96°C for 1 min, 96°C for 10 s, 50°C for 5 s, and 60°C for 4 min, followed by a 4°C infinite hold. The Sephadex G50 superfine (Sigma–Aldrich) was used to remove unincorporated dye terminators by washing the column with 300 μL of distilled water and centrifuging at 1,500 *g* for 3 min before applying the sample to the column. The sequencing reaction was performed using the forward and reverse primers FD1 and RP2. Forward and reverse sequencing were performed using Big Dye Terminator version 3.1 cycle sequencing kit (Applied Biosystems, Foster City, CA). Sequence trimming and assembly were performed using DNA baser. Then, the sequences were submitted to Basic Local Alignment Search Tool (BLAST) (Altschul et al., 1990) and ClustalW was used for multiple alignment with available *16S rRNA* sequences in GenBank (National Center for Biotechnology Information NCBI, Bethesda, MD, USA). Phylogenic relationships were performed by Mega 11 software (Stecher et al., 2020; Tamura et al., 2021). The phylogenetic tree was built using the neighbor-joining method (Saitou and Nei, 1987) with statistic method of maximum likelihood, taking into account the correction of Jukes and Cantor (1969). Tree topology was evaluated by bootstrap analyses (1,000 replicates) (Felsenstein, 1985). The cutoff for species identification was 98.8%. The sequence data were deposited at the GenBank database under the following accession numbers PsT-04c (OP677775), PsT-94s (OP677776), PsT-116 (OP677777), PsT-124 (OP677861), PsT-130 (OP677782), and BaT-68s (OP677783).

### 2.7 Data analysis

Values are means and standard deviation of three or more than three replicates. Statistical analyses were conducted using SPSS software package version 22 for Microsoft Windows. The data obtained were examined through analysis of variance (ANOVA), to test the statistical differences. Means were compared at the 5% significance level using Tukey’s post-hoc test, to detect statistically significant differences between treatments. Principal component analysis (PCA) was performed using GraphPad Prism 9 software.

## 3 Results

### 3.1 Isolation of bacteria and screening for P, K, and Zn solubilizing activity and IAA production

**Figure 1:**
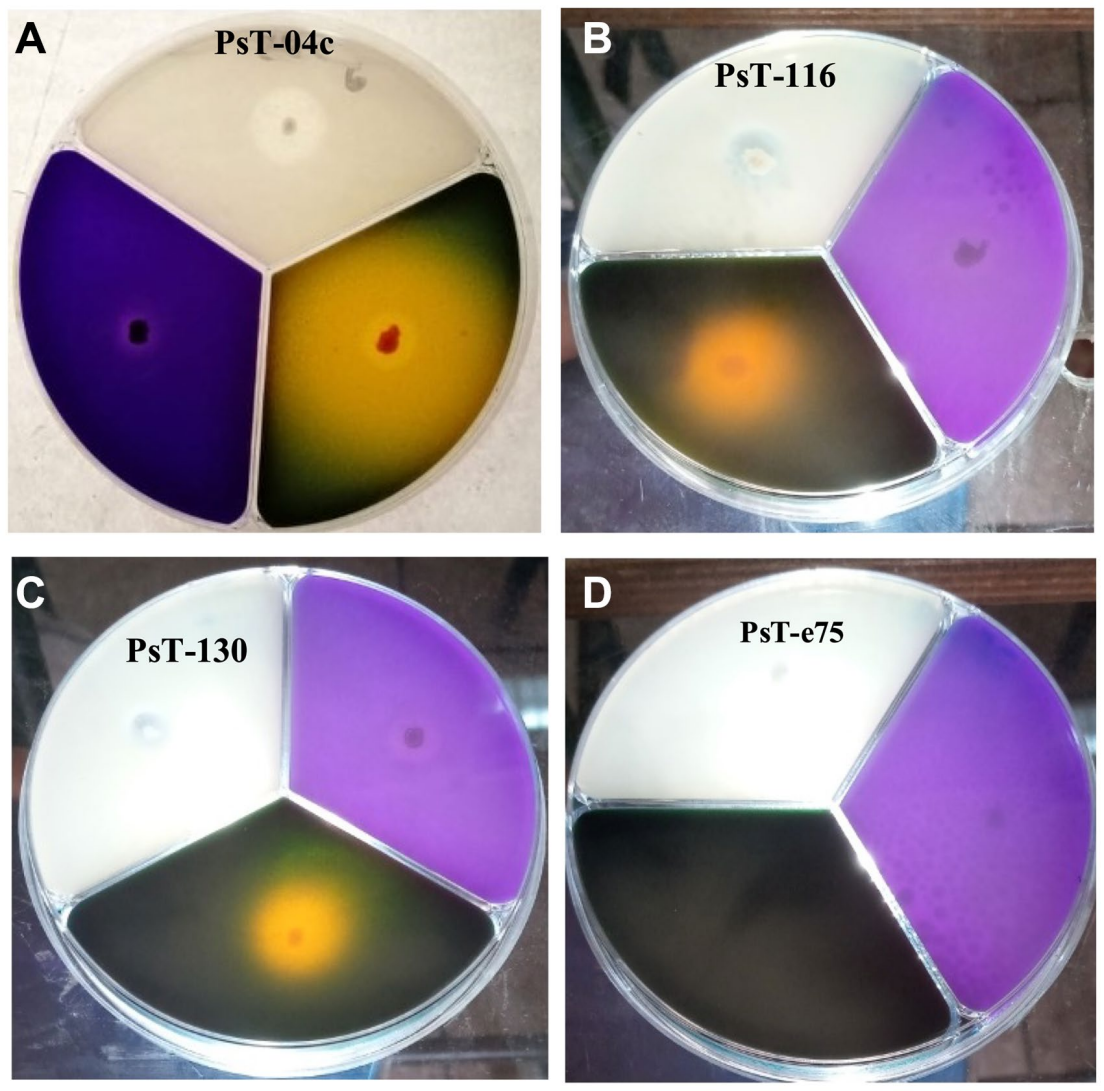
Solubilization of phosphorus, potassium, and zinc insoluble forms on NBRIP (purple medium), Alexandrov (green medium), and Bunt and Rovira media (translucid medium) respectively by three solubilizing strains PsT-04c **(A)**, PsT-116 **(B)**, and PsT-130 **(C)** compared with a non- solubilizing strain PsT-e75 **(D)**.

**Figure 2:**
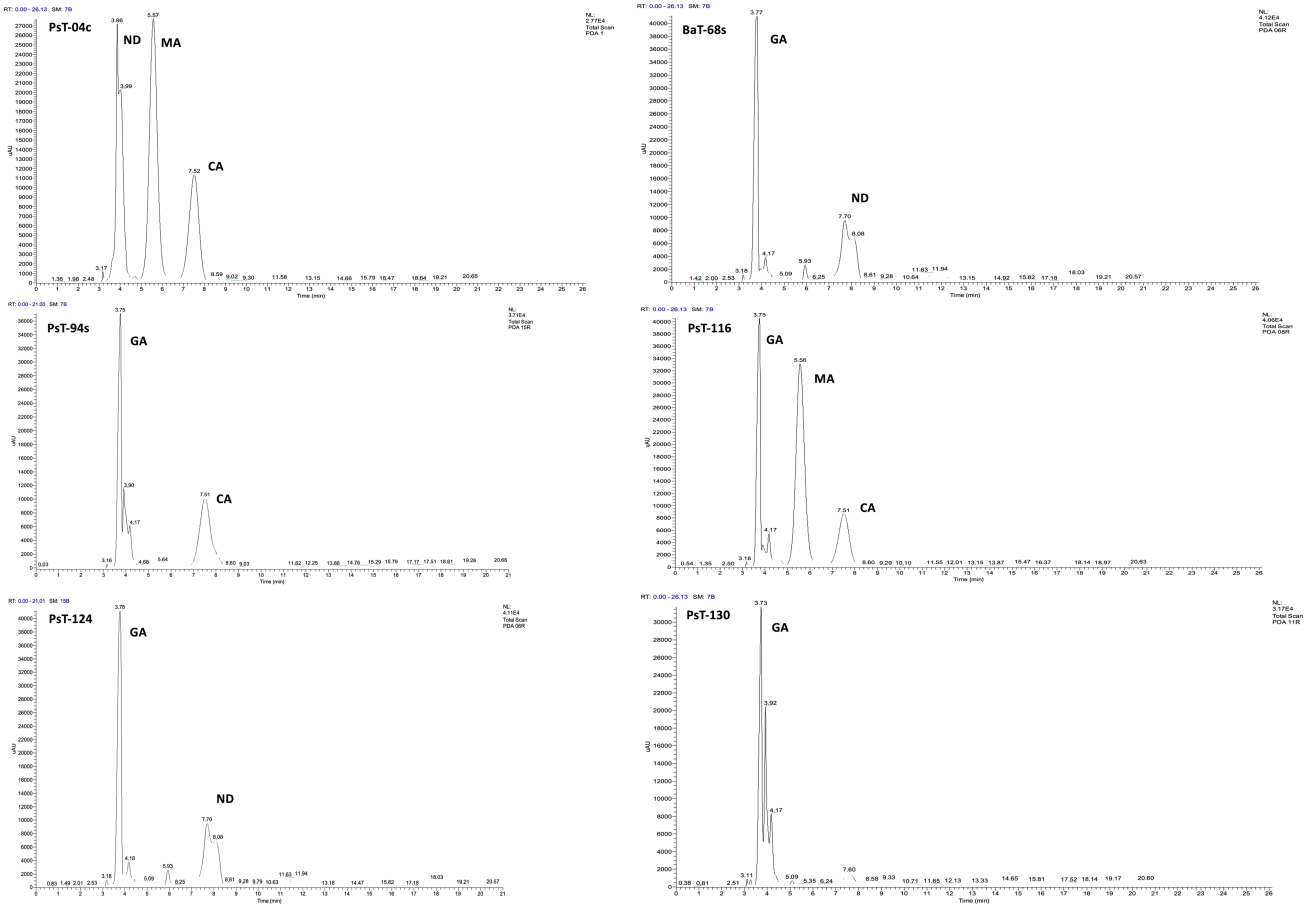
C18-HPLC profiles of the organic acids secreted by the six selected bacterial isolates PsT-04c, BaT-68s, PsT-94s, PsT-116, PsT-124, and PsT-130. GA, gluconic acid; CA, citric acid; MA, maleic acid; ND, not determined.

In the present study, we screened isolated bacteria for effective phosphate solubilization, and we investigated *in vitro* PGP traits and their potential as tomato plant growth promoters. The primary isolation of bacteria from tomato rhizosphere soil was performed on King B medium. In total, 100 different bacterial strains were isolated. Among these bacteria, those which developed yellow-green fluorescent pigment under UV light (365 nm) are putatively considered, belonging to the fluorescent pseudomonads. The isolates were further screened for phosphate solubilizing ability. Out of 100 isolates tested on NBRIP agar medium, 16 isolates (PsT-04c, PsT-11b, PsT-35b, PsT-63k, BaT-68s, PsT-77, PsT-82k, PsT-88k, BaT-91k, PsT-92k, PsT-94s, PsT-96k, PsT-98k, PsT-116, PsT-124, and PsT-130) were able to solubilize phosphate on NBRIP (Table 1). These isolates exhibited clear halo zones on NBRIP after 24–72 h of incubation at 28°C (Figure 1). In NBRIP broth, the growth of these isolates was accompanied with increased amount of released P ranging from 73.22 to 195.42 mg mL^−1^. The maximum amount was showed by isolate PsT-94s, while the lowest amount was detected in isolate PsT-63k. All isolates lowered pH of the medium compared with control. All isolates brought the pH value of the medium below 6. The pH of the medium at initial conditions ranged between 6.7 and 6.9 and dropped to 3.22 as the lowest value for the isolate PsT-130 after 48 h, suggesting acidification as the main mechanism for solubilization. Organic acids were detected by HPLC/MS in NBRIP cultures of six bacterial isolates (Figure 2 and Supplementary Figure S1), showing highest phosphate solubilization (isolates PsT-04c, BaT-68s, PsT-94s, PsT-116, PsT-124, and PsT-130). Retention times for all isolates were compared with those of standards. The main organic acids detected were gluconic acid, maleic acid, and citric acid (Figure 2). The rest of peaks were not determined in this investigation.

**Table 1:** Fluorescence under UV, phosphate, potash, and zinc solubilization and IAA production by 16 bacterial isolates.

Isolate	Fluorescence under UV	P solubilization index (PSI)*	P released** (mg L^−1^)	pH at 48 h*	K solubilization index (KSI) *	Zn solubilization index (ZSI)*	IAA (μg mL^−1^)**
Control	−	−	−	6.82 ± 0.01^a^	−	−	0.72 ± 0.51^j^
PsT-04c	+	4.34 ± 2.32^ab^	155.09 ± 1.82^b^	3.63 ± 0.02^g^	4.46 ± 1.89^abc^	4.55 ± 0.97^ab^	84.96 ± 1.53^bc^
PsT-11b	+	1.08 ± 0.06^c^	90.61 ± 0.24^g^	4.15 ± 0.03^e^	2.55 ± 0.10^bcdef^	1.00 ± 0.00^d^	50.96 ± 2.17^h^
PsT-35b	+	1.44 ± 0.33^c^	116.01 ± 0.29^e^	5.11 ± 0.01^b^	2.70 ± 0.71^bcdef^	1.59 ± 0.23^d^	2.05 ± 0.44^j^
PsT-63 k	+	1.20 ± 0.06^c^	73.22 ± 0.15^i^	4.96 ± 0.00^b^	1.30 ± 0.15^f^	1.00 ± 0.00^d^	73.80 ± 2.09^d^
BaT-68s	−	3.16 ± 0.76^bc^	114.24 ± 6.13 ^e^	3.61 ± 0.04^g^	4.92 ± 1.31^ab^	4.38 ± 0.48^abc^	81.70 ± 1.91^c^
PsT-77 k	+	1.16 ± 0.10^c^	113.45 ± 0.26^e^	4.92 ± 0.05^b^	2.50 ± 1.08^bcdef^	1.75 ± 0.78^cd^	58.63 ± 1.24^g^
PsT-82 k	+	2.96 ± 1.48^bc^	99.74 ± 0.13^f^	4.95 ± 0.02^b^	4.34 ± 1.82^abcd^	2.13 ± 0.53^bcd^	67.80 ± 1.94^e^
PsT-88 k	−	1.33 ± 0.16^c^	113.53 ± 0.37^e^	4.80 ± 0.02^c^	2.13 ± 0.09^cdef^	1.50 ± 0.10^d^	95.63 ± 1.39^a^
BaT-91 k	−	2.75 ± 1.12^bc^	77.74 ± 1.70^h^	3.67 ± 0.00^g^	3.81 ± 0.89^bcdef^	1.00 ± 0.00^d^	66.88 ± 0.93^ef^
PsT-92 k	−	1.13 ± 0.059^c^	95.49 ± 2.38^f^	3.86 ± 0.01^f^	1.78 ± 0.25^def^	1.00 ± 0.00^d^	2.96 ± 1.42^j^
PsT-94s	+	3.38 ± 2.35^bc^	195.42 ± 1.60^a^	4.34 ± 0.04^d^	4.87 ± 0.14^abcd^	4.71 ± 1.17^ab^	73.05 ± 1.48^d^
PsT-96 k	+	1.19 ± 0.13^c^	77.28 ± 0.31^hi^	4.95 ± 0.02^b^	4.14 ± 0.57^abcde^	2.95 ± 0.49^bcd^	58.55 ± 1.76^g^
PsT-98 k	+	1.27 ± 0.12^c^	73.99 ± 4.94^hi^	4.94 ± 0.02^b^	1.56 ± 0.62^ef^	1.71 ± 0.30^cd^	12.05 ± 2.91^i^
PsT-116	+	4.73 ± 2.02^ab^	133.32 ± 0.26^c^	4.92 ± 0.02^b^	4.31 ± 1.59^abcd^	3.48 ± 3.02^abcd^	85.96 ± 2.13^b^
PsT-124	+	3.47 ± 0.35^bc^	123.32 ± 0.20^d^	3.61 ± 0.04^g^	1.25 ± 0.00^f^	3.61 ± 0.42^abcd^	76.13 ± 0.73^d^
PsT-130	+	6.69 ± 0.41^a^	151.20 ± 0.25^b^	3.22 ± 0.06^h^	6.60 ± 1.5^a^	5.93 ± 1.16^a^	63.46 ± 4.10^f^

*The values represent the mean values ± SD of six replicates. **The values represent the mean values ± SD of four replicates. Means followed by the same letters are not significantly different at *p* < 0.05.

Most of the selected bacterial isolates for phosphate solubilizing ability also solubilized insoluble forms of potash and zinc and produced IAA (Figure 1 and Table 1). The isolates PsT-11b, PsT-63k, BaT-91k, and PsT-92k did not solubilize insoluble form of zinc. Isolates PsT-04c, PsT-94s, BaT-68s, and PsT-130 showed the highest potassium solubilization with index values of 4.46, 4.87, 4.92, and 6.60, respectively. The selected bacterial isolates showed significant levels of hormone IAA production (Table 1). The IAA amounts exceeded 80 μg mL^−1^ for several isolates. The six isolates PsT-88 k, PsT-116, PsT-04c, BaT-68s, PsT-124, PsT-63 k, and PsT-94s produced 95.63, 85.96, 84.96, 81.70, 76.13, 73.80, and 73.05 μg mL^−1^ of IAA, respectively, while only less amount was produced by isolates PsT-98 k (12.05 μg mL^−1^), PsT-92 k (2.96 μg mL^−1^), and PsT-35b (2.05 μg mL^−1^).

### 3.2 Plant growth-promoting effects on tomato

#### 3.2.1 Effects on growth parameters

The six bacterial isolates, which showed the highest phosphate solubilization and IAA production abilities (isolates PsT-04c, BaT-68s, PsT-94s, PsT-116, PsT-124, and PsT-130), were selected for further experiments in order to test their abilities to show positive effects on seed germination and successfully colonize tomato rhizosphere (Table 2). The results showed that the germination rate of the seeds was improved in the presence of the isolates. Results of the effect of selected isolates combined with RP on seed germination showed that all treatments with bacterial isolates and RP had a significant positive effect on the germination rate at *p* < 0.05 compared with the controls consisting of seeds treated only with RP (RP amended, bacteria-free seeds). The maximum increase was obtained by the isolates PsT-04c and PsT-130 (Table 2). Greenhouse experiments were conducted by using these six isolates, and shoot and root length and fresh and dry weight were measured at the end of the experiment (Table 3). The maximum effect on shoot length and weight was showed by the isolates PsT-04c, BaT-68s, and PsT-94s, while the maximum positive effect on roots was showed by the isolates PsT-116 and BaT-68s. The results showed that the shoot length was increased by 45.51 and 43.25 % when seedlings were treated by the isolates PsT-04c and PsT-94s, respectively, while the root length reached up to 54.52% by the isolate PsT-116. Shoot and root fresh weight increased up to 63.68 and 72.96 %, respectively, while dry weight increased by 56.26 and 78.13%, respectively.

**Table 2:** Seed germination, rhizosphere colonization, growth from root tissues, and on root exudates displayed by the six bacterial isolates.

**Treatment**	Seed germination (%)^*^	**Rhizosphere colonization** **(x10**^**6**^**cfu g**^**−1**^)^**^	**Growth from root tissues** **(x10**^**5**^**cfu g**^**−1**^**roots)**	**Growth on root exudates** **(x10**^**6**^**cfu g**^**−1**^**roots)**
Control	80 ± 3.00^d^	–	–	–
PsT-04c	100 ± 0.00^a^	2.0 ± 1.05^a^	6.00	30.00
BaT-68s	98 ± 1.00^ab^	1.21 ± 0.28^a^	1.00	0.05
PsT-94s	90 ± 4.35^c^	1.30 ± 0.62^a^	2.00	0.01
PsT-116	95 ± 1.00^abc^	2.75 ± 0.77^a^	1.00	1.00
PsT-124	94 ± 1.00^bc^	2.34 ± 1.37^a^	3.00	0.02
PsT-130	100 ± 0.00^a^	1.63 ± 0.75^a^	7.00	6.00

*The values represent the mean values ± SD, the experiment was performed with three replicates (each consisted of 50 seeds). **The values represent the mean values ± SD of three replicates (each consisted of six seedlings).

**Table 3:** Plant growth promotion displayed by selected bacterial isolates: on tomato (calvi cv.): effects on shoot and root length and fresh and dry weight, percentage between parenthesis correspond to increase (%) in shoot and root length and fresh weight.

**Treatment**	Shoot^***^				Root^***^	
	**Length (cm)**	**Fresh weight (g)**	**Dry weight (g)**	**Length (cm)**	**Fresh weight (g)**	**Dry weight (g)**
Control	51.04 ± 0.75^c^	26.65 ± 1.55^b^	4.23 ± 0.23^b^	20.86 ± 4.21^c^	10.69 ± 1.99^b^	1.92 ± 0.87^b^
PsT-04c	74.27 ± 2.72^a^ (45.51)	43.62 ± 3.48^**a**^ (63.67)	6.22 ± 1.03^a^	29.07 ± 4.23^ab^ (39.38)	17.72 ± 1.80^a^ (65.76)	3.30 ± 0.34^ab^
BaT-68s	72.00 ± 5.29^ab^ (41.18)	41.75 ± 6.35^a^ (56.66)	6.05 ± 2.00^ab^	30.60 ± 4.23^ab^ (46.71)	13.97 ± 3.39^ab^ (30.74)	2.74 ± 1.06^ab^
PsT-94s	73.06 ± 3.89^a^ (43.25)	41.35 ± 4.04^a^ (55.17)	5.97 ± 0.59^ab^	27.14 ± 1.06^bc^ (30.14)	18.49 ± 2.96^a^ (72.97)	3.42 ± 1.10^a^
PsT-116	66.71 ± 3.67^b^ (30.81)	43.31 ± 3.62^a^ (62.51)	6.61 ± 0.90^a^	32.23 ± 4.62^a^ (54.52)	17.03 ± 2.73^a^ (59.38)	2.71 ± 0.90^ab^
PsT-124	65.94 ± 3.15^b^ (29.30)	39.93 ± 4.23^a^ (49.82)	5.69 ± 1.30^ab^	28.17 ± 4.23^ab^ (35.07)	14.06 ± 3.38^ab^ (31.60)	2.26 ± 0.65^ab^
PsT-130	68.51 ± 3.59^ab^ (34.34)	40.91 ± 4.77^a^ (53.52)	6.57 ± 0.76^a^	28.19 ± 4.23^ab^ (35.14)	14.07 ± 3.37^ab^ (31.65)	2.32 ± 0.69^b^

***The values represent the mean values ± SD of 30 plants (the whole experiment was performed in three replicates). Means followed by the same letters are not significantly different at *p* < 0.05.

#### 3.2.2 Effects on mineral, sugars, proteins, and chlorophyll contents and chlorophyll fluorescence

**Figure 3:**
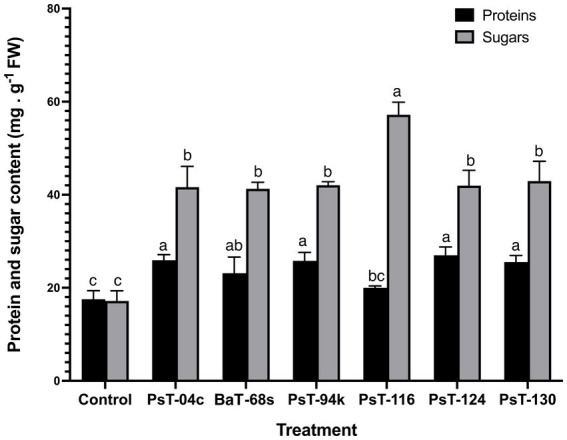
Effect of treatments by selected bacterial isolates on protein and sugar contents of tomato seedlings after 60 days. Different letters above the bars indicate the differences are significant at *P* < 0.05.

The bacterial isolates were further investigated for their abilities to enhance nutrient contents of protein, sugars, calcium (Ca), sodium (Na), potassium (K), phosphorus (P), and chlorophyll. Sugar contents enhanced by up to three-fold with the isolate PsT-116, while protein contents enhanced by 47.77, 46.90, and 45.55%, respectively, by isolates PsT-04c, PsT-94s, and PsT-130 (Figure 3). In comparison to the control, all bacterial isolates showed significant increases in K, Ca, and Na contents of the shoot and root, with the maximum increase by isolate BaT-68s for root K, and PsT-94s for root Na and Ca (Table 4). The maximum amounts of shoot Ca and K were detected following treatment with strain PsT-124 while the maximum amount of Na was obtained by isolates PsT-124 and PsT-130. The results also showed a significant increase in P content in leaves by up to three-fold in seedlings treated with PsT-94s, in comparison to non-inoculated control. Total chlorophyll content increased by up to 43–47% in seedlings treated with the isolates PsT-04c and PsT-94s, respectively, while Chl a and Chl b increased by 40 and 55%, following treatment by the isolate PsT-94s. The maximum increases in Chl a and Chl b were shown respectively by the isolate BaT-68s (47%) and PsT-04c (76%). No significant changes in chlorophyll fluorescence (photosynthetic quantum yield: Fv/Fm) were observed after 60 days of culture (Table 5).

**Table 4:** Effect of inoculation by selected bacterial isolates on phosphorus content of tomato leaves and shoot and root cation element contents: potassium (K), calcium (Ca), and sodium (Na) after 60 days.

**Treatment**	Leaf phosphorus	**Shoot**			**Root**		
	**P** **(μg mg**^**−1**^**DW)**	**K** **(μg mg**^**−1**^**DW)**	**Na** **(μg mg**^**−1**^**DW)**	**Ca** **(μg mg**^**−1**^**DW)**	**K** **(μg mg**^**−1**^**DW)**	**Na** **(μg mg**^**−1**^**DW)**	**Ca** **(μg mg**^**−1**^**DW)**
Control	51.71 ± 5.52^a^	236.29 ± 9.58^a^	46.46 ± 5.84^a^	228.21 ± 7.48^a^	201.04 ± 1.00^a^	116.80 ± 0.89^bc^	152.28 ± 6.53 ^a^
PsT-04c	140.31 ± 9.87^bc^	347.96 ± 15.31^cd^	129.44 ± 7.47^cd^	384.75 ± 0.90^c^	200.29 ± 3.84^a^	94.82 ± 6.11^a^	203.25 ± 3.47 ^b^
BaT-68s	145.31 ± 8.66^bc^	357.26 ± 0.87^cd^	107.66 ± 0.60^b^	376.56 ± 0.95^c^	277.46 ± 10.25^c^	118.84 ± 1.28^c^	167.78 ± 6.87 ^a^
PsT-94s	164.34 ± 3.62^c^	317.11 ± 11.38^bc^	112.33 ± 0.56^bc^	311.68 ± 4.87^b^	227.37 ± 5.61^ab^	166.04 ± 4.17^d^	214.25 ± 5.09 ^b^
PsT-116	110.56 ± 8.79^b^	301.33 ± 3.28^b^	127.11 ± 2.15^bcd^	314.43 ± 3.94^b^	237.53 ± 1.65^b^	96.85 ± 3.98^a^	212.62 ± 4.64^b^
PsT-124	137.62 ± 5.91^bc^	376.38 ± 0.72^d^	136.11 ± 4.38^d^	394.69 ± 2.56^c^	245.16 ± 6.17^b^	99.50 ± 3.29^ab^	204.58 ± 2.31^b^
PsT-130	125.35 ± 7.06^b^	367.96 ± 4.82^d^	134.28 ± 5.08^d^	386.22 ± 4.80^c^	201.50 ± 5.36^a^	104.82 ± 3.57^abc^	205.71 ± 3.53^b^

The values represent the mean values ± SD of three replicate. Means followed by the same letters are not significantly different at *p* < 0.05.

**Table 5:** Effect of inoculation by selected bacteria on the chlorophyll content of tomato leaves (expressed as mg/dry weight) and chlorophyll fluorescence (Fv/Fm).

**Treatment**	**Chlorphyll a** **(mg g**^**−1**^DW)^*^	**Chlorphyll b** **(mg g**^**−1**^DW)^*^	**Total Chlorphyll** **(mg g**^**−1**^DW)^*^	Photosynthetic quantum yield (Fv/Fm)^**^
Control	9.24 ± 0.79^a^	4.71 ± 0.45^a^	10.02 ± 0.91^a^	0.76 ± 0.200^ab^
PsT-04c	11.06 ± 0.18^ab^	8.29 ± 0.36^d^	14.33 ± 0.38^bc^	0.77 ± 0.038^ab^
BaT-68s	13.63 ± 0.92^c^	5.97 ± 0.15^ab^	13.92 ± 0.66^bc^	0.75 ± 0.009^ab^
PsT-94s	12.92 ± 0.39^bc^	7.30 ± 0.38^cd^	14.64 ± 0.48^c^	0.730 ± 0.011^a^
PsT-116	11.14 ± 0.67^ab^	5.79 ± 0.07^ab^	12.18 ± 0.02^ab^	0.74 ± 0.054^ab^
PsT-124	10.78 ± 0.25^ab^	6.20 ± 0.22^bc^	12.34 ± 0.17^b^	0.79 ± 0.194^b^
PsT-130	10.00 ± 0.04^a^	6.60 ± 0.06^bc^	12.16 ± 0.04^ab^	0.77 ± 0.050^ab^

^*^The values represent the mean values ± SD of four replicates. ^**^The values represent the mean values ± SD of seven replicates. Means followed by the same letters are not significantly different at *p* < 0.05.

#### 3.2.3 Principal component analysis

**Figure 4:**
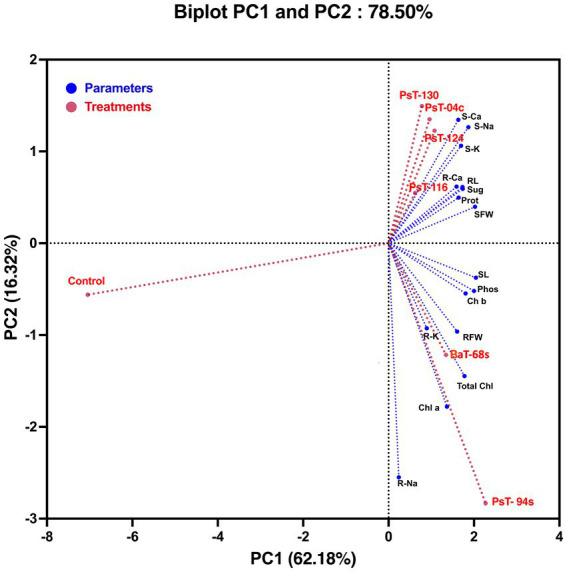
Principal component analysis (PCA) of tomato plants subjected to phosphate solubilizing bacteria. The variables (agro-morphological, physio-biochemical, and minerals including phosphorus) are presented in blue while bacterial treatments are presented in red. Control: absence of the phosphate solubilizing bacteria, PsT-04c, PsT-94s, PsT-116, PsT-124, and PsT-130: treatments with phosphate solubilizing *Pseudomonas* sp. isolates. BaT-68s: treatment with phosphate solubilizing *Bacillus* isolate. SL, shoot Lenght; RL, root length; SFW, shoot fresh weight; RFW, root fresh weight; S-Ca, shoot calcium content; S-Na, shoot sodium content; S-K, shoot potassium content; R-Ca, root calcium content; R-Na, root sodium content; R-K, root potassium content; Phos, plant leaf phosphorus; Sug, total soluble sugar content; Prot, protein content; Chl a, chlorophyll a; Chl b, chlorophyll b; Total Chl, total chlorophyll.

To assess the contributions of each parameter to phosphate solubilizing bacteria-treated tomato plants compared with control plants, we performed a principal component analysis using plant growth and physiological parameters. The principal component analysis showed that bacterial treatments (red) and variables (blue) were associated with two top PCs, accounting for 78.50% of the total variation in traits under greenhouse conditions (Figure 4). PC1 explained 62.18% of the total variation and was strongly influenced by morphological and biochemical parameters, while PC2 accounted for 16.32% of the total variation and was strongly associated with physiological photosynthetic parameters. The PCA showed a positive correlation between applied phosphate solubilizing bacteria and growth parameters, photosynthetic pigments, and protein and sugar contents, which were positively correlated with each other and confirmed the positive impact of the used bacteria on growth, physiology, biochemistry, and nutrition parameters. In addition, PCA showed that all applied treatments were separated from their controls. Agro-morphological (shoot fresh weight and root fresh weight), physio-biochemical (total soluble sugar, protein, chlorophyll a, chlorophyll b and total chlorophyll contents), shoot and root minerals (Na, K, and Ca) and leaf phosphorus parameters grouped the applied treatments into three main groups; the best responses in terms of higher growth and physio-biochemical parameters were shown by treatments with strains PsT-04c, PsT-116, PsT-124, and PsT-130 (right side of the first axis PC1, upper panel). In addition, treatments with strains PsT-04c and BaT-68s (right side of the first axis, lower panel) resulted in better efficient photosynthetic system and phosphorus nutrition. In contrast, the control treatment without inoculation showed lower growth in both aboveground and root parts, in addition to lower mineral nutrition.

### 3.3 Protection of tomato against *Clavibacter michiganensis* subsp. *michiganensis (Cmm)* by induced systemic resistance

#### 3.3.1 Ability of bacteria to grow inside root tissues and on root exudates

Bacterial growth in the rhizosphere depends on their ability to positively interact with the root system, since rhizosphere represents the contact point of plant with major soil microorganisms. In this study, all the six isolates showed abilities to develop in root tissues and on root exudates. In root tissues, their numbers were: PsT-04c: 6.10^5^, BaT-68s: 10^5^, PsT-94s: 2. 10^5^, PsT-116: 10^5^, PsT-124: 3.10^5^, and PsT-130: 7.10^5^ cfu g^−1^ roots. Moreover, the two isolates Pst-04c and Pst-130 showed the highest ability to grow on tomato root exudates: 3.10^7^ and 6.10^6^ cfu g^−1^ roots, respectively (Table 2). Hence, the isolates PsT-04c and PsT-130 were selected and used in greenhouse experiments for application as biocontrol agents against the causal agent of bacterial canker, *Clavibacter michiganensis* subsp. *michiganensis* (*Cmm),* by enhanced induced resistance.

#### 3.3.2 Protection against tomato bacterial canker

For biocontrol experiments by resistance induction, we used an aggressive isolate of *Cmm* and the two isolates PsT-04c and PsT-130 in greenhouse, and we performed the following experiments (i) non-bacterized, uninfected control seedlings, (ii) seedlings infected with *Cmm,* and (iii) bacterized seedlings with the *Pseudomonas* isolates PsT-04c and PsT-130 and infected with *Cmm* 1 week after the first bacterization and bacterized again 20 days after *Cmm* inoculation.

**Figure 5:**
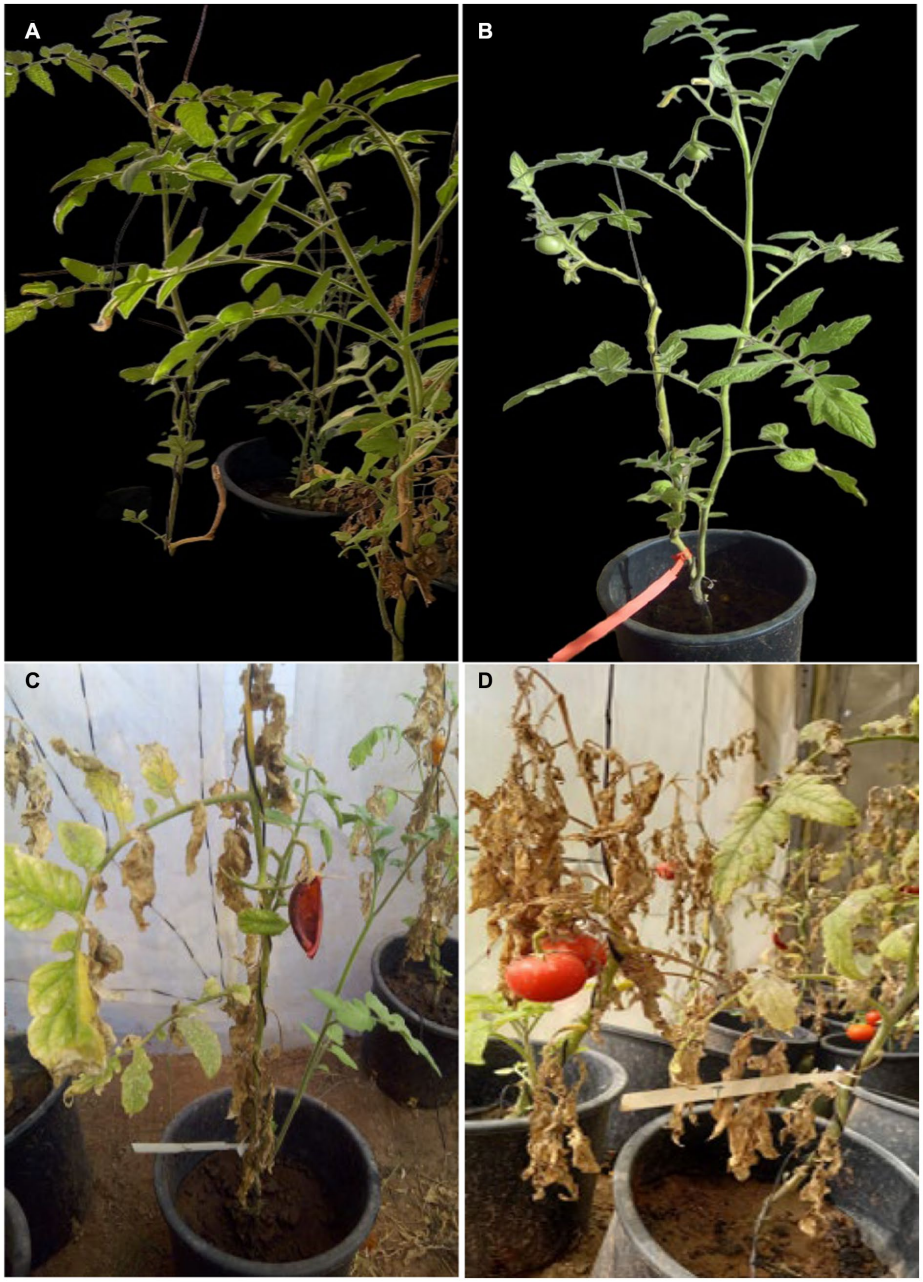
Representative pictures showing biocontrol of tomato bacterial canker after 60 days. **(A)** Control tomato seedling, **(B)** Tomato seedlings inoculated with the *Pseudomonas* isolate PsT-04c and susbesquently infected with *Clavibacter michiganensis,* **(C,D)** Decaying tomato seedlings infected with *Cmm* showing bacterial canker symptoms.

**Figure 6:**
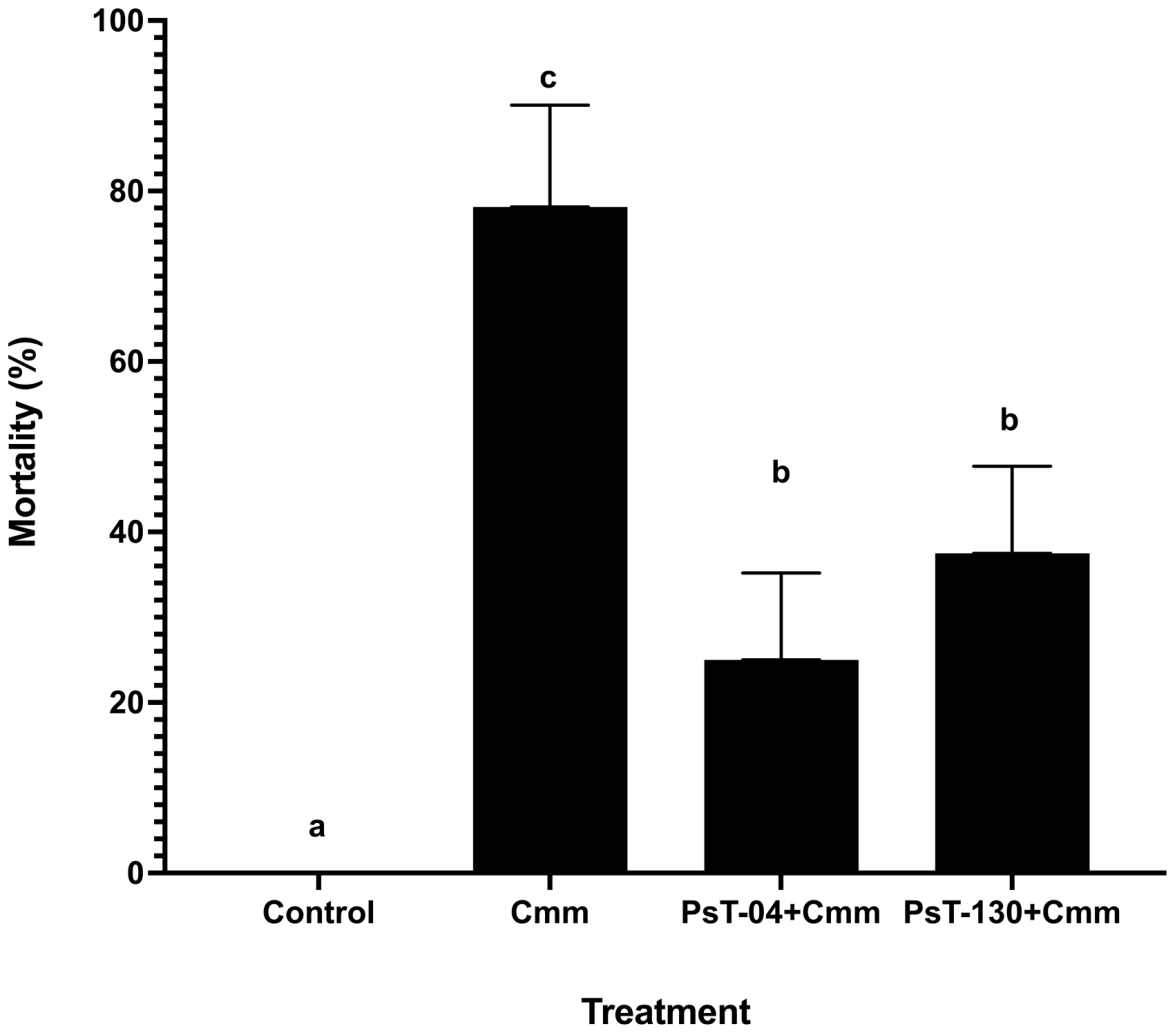
The mortality rate recorded after 120 days in tomato seedling treated with water (negative control), in tomato seedlings artificially infected with *Cmm* (positive control), and in tomato seedlings inoculated with isolates PsT-04c and PsT-130 before infection by *Cmm*. Different letters above the bars indicate that the differences are significant at *p* < 0.05.

Figure 5 shows symptoms on infected seedlings which resulted in plant death compared with control and bacterized seedlings. Artificial infection of tomato seedlings by the pathogen *Cmm* led to a high mortality rate of approximately 80% compared with the control. Treatments with *Pseudomonas* isolates PsT-04c and PsT-130 led to a significant reduction in the mortality of tomato plants infected with *Cmm* (Figure 6). At the end of experiment (120 days), no mortality was detected in the negative control, which received sterilized water. In the bacterized seedlings by Pst-04c PsT-130, bacterial canker symptoms were very limited to the leaves. Moreover, appearance of symptoms in the *Cmm*-infected seedlings was detected after 40 days to 2 months, while appearance of the first symptoms was delayed by one month in the bacterized seedlings by PsT-130 and 40 days in those bacterized by PsT-04c. After 120 days, bacterized seedlings resulted in a protection rate of 75% by PsT-04c and 64% by PsT-130 (Figure 6).

#### 3.3.3 Ability to induce systemic resistance mechanisms

##### 3.3.3.1 Total soluble phenolic compounds

**Figure 7:**
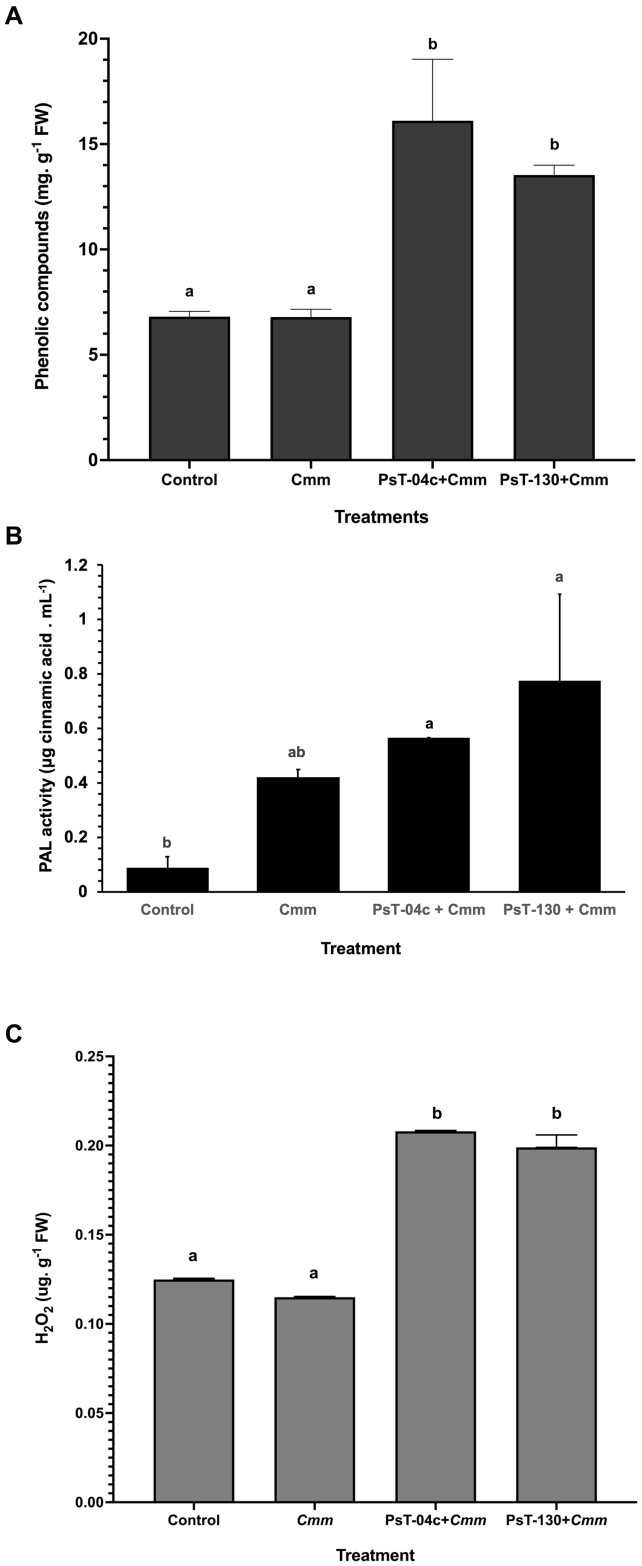
Mechanisms of defense induced in bacterized tomato seedlings by the two *Pseudomonas* isolates PsT-04c and PsT-130 compared with negative control and *Cmm*-infected seedlings. **(A)** Phenolic compounds contents, **(B)** Phenylalanine ammonia-lyase (PAL) activity, and **(C)** H_2_O_2_ content. *Cmm*: *Clavibacter michiganensis* positive control, *Cmm* + PsT-04c, and *Cmm* + PsT-130: seedlings treated, respectively, with PsT-04c and PsT-130 and post challenged with *Cmm*. Different letters above the bars indicate that the differences are significant at *p* < 0.05.

After 43 days of infection by *Cmm*, corresponding to the appearance of bacterial canker symptoms in infected plants, soluble phenolic compounds were measured in *Pseudomonas*-treated seedlings compared with the control and *Cmm*-infected seedlings. The treatment with *Pseudomonas* resulted in an increase in the total root-soluble phenolic compounds of the treated tomato (Figure 7A). Content of total soluble phenolic compounds was 6.81 mg g^−1^ FW in the untreated plants. No significant differences were found in *Cmm*-infected plants (6.79 mg g^−1^ FW) compared with the control. Their content increased in bacteria-treated plants and reached 16.11 and 13.53 mg g^−1^ FW, respectively, by strains PsT-04c and PsT-130. These levels represented 5.86 times for PsT-04c and 4.42 times for PsT-130 in comparison to the control.

##### 3.3.3.2 Phenylalanine ammonia-lyase

After 43 days of infection by *Cmm*, corresponding to the appearance of bacterial canker symptoms in infected plants, phenylalanine ammonia-lyase (PAL) contents were measured in *Pseudomonas*-treated seedlings compared with the control and *Cmm*-infected seedlings. The results of phenylalanine ammonia-lyase (PAL) analysis showed that isolates PsT-04c and PsT-130 enhanced PAL activity. After one month of the second bacterization, seedlings treated by PsT-04c and PsT-130 and subsequently infected by *Cmm* showed higher PAL amount (respectively 0.563 and 0.773 μg mL^−1^) in comparison to the control (0.088 μg mL^−1^). PAL activities in PsT-04c and PsT-130 treated seedlings were 6.37 and 8.76 times higher than the control. *Cmm*-infected tomato plants showed only 0.42 μg mL^−1^ representing an increase by 4.75 times in comparison to the control (Figure 7B).

##### 3.3.3.3 Hydrogen peroxide (H_2_O_2_) content

After 43 days of infection by *Cmm*, corresponding to the appearance of bacterial canker symptoms in infected plants, hydrogen peroxide (H_2_O_2_) contents were measured in *Pseudomonas*-treated seedlings compared with the control and *Cmm*-infected seedlings. Treatments with *Pseudomonas* isolates PsT-04c and PsT-130 led to a significant enhancement of H_2_O_2_ in the tomato plants infected with *Cmm* compared with the control, 1month after the second bacterization (Figure 7C). Isolates PsT-04c showed the highest amount of H_2_O_2_ of 0.208 μg g^−1^ FW compared with the control (0.115 μg g^−1^ FW), while no significant changes were detected in plants infected with *Cmm* (0.125 μg g^−1^ FW) compared with the control.

### 3.4 Molecular identification of the bacterial isolates

**Figure 8:**
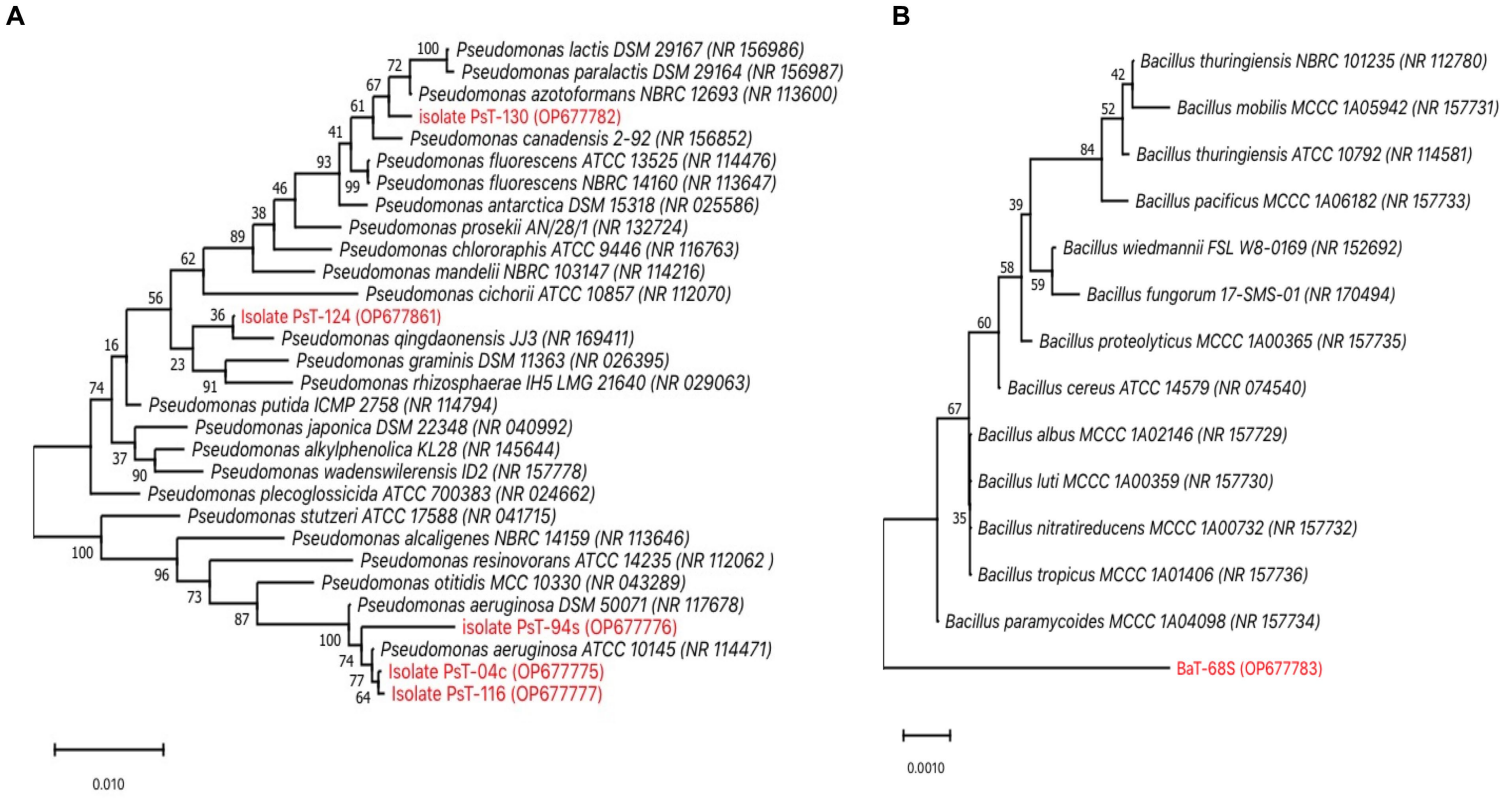
Unrooted consensus phylogenetic trees reconstructed by the neighbor-joining method (Saitou and Nei, 1987) based on almost-complete *16S rRNA* gene sequences (1,500 bp). **(A)** phylogenetic relationships between five fluorescent Pseudomonad isolates (PsT-04c, PsT-94s, PsT-116, PsT-124, and PsT-130) and 25 closely related *Pseudomonas* type species, **(B)** phylogenetic relationships between BaT-68s and 13 closely related *Bacillus* species. Closest species were derived using NCBI BLAST search tool (Altschul et al., 1990). The GenBank accession numbers of species are placed in parenthesis. Sequences were aligned using Muscle, and Evolutionary analyses were conducted in MEGA 11 software (Stecher et al., 2020; Tamura et al., 2021). Bootstrap support values: percentage of replicate trees in which the associated taxa clustered together in the bootstrap test (1,000 replicates) are shown above the branches (Felsenstein, 1985). The evolutionary distances are computed using the Maximum Composite Likelihood method (Tamura et al., 2004) and are in the units of the number of base substitutions per site. The scale bar represents nucleotide substitutions per site. There were 1,558 positions for *Pseudomonas* and 1,580 for *Bacillus* in the final dataset. Isolates PsT-04c, PsT-94s, and PsT-116 shared >99% sequence identity with the type strains of *Pseudomonas aeruginosa.* Isolate PsT-124 shared >99% sequence identity with *P. qingdaonensis.* Isolate PsT-130 showed high similarity level to *P. azotoformans*, isolate BaT-68s shared >99% sequence identity with *Bacillus paramycoides*.

The six isolates were identified using phenotypic and genotypic techniques (Bossis et al., 2000). API 20NE identification system and other biochemical traits showed that all the strains were different (Supplementary Table S1). All isolates were gram-negative except BaT-68s, unicellular bacilli with circular white to yellow colonies. All the five fluorescent isolates PsT-04c, PsT-94s, PsT-116, PsT-124, and PsT-130 were oxidase-positive, suggesting their belonging to the fluorescent pseudomonad group. They were also positive for Levan, except for PsT-94s, and tween, except for PsT-124. All strains were neither able to produce H_2_S nor to assimilate citrate. Furthermore, they were able to hydrolyze gelatin, except for PsT-124. Based on *16SrRNA* gene sequences, the molecular identification revealed that all isolates shared high sequence similarity (>99%) with species from the genus *Pseudomonas*. Moreover, the phylogenetic analysis constructed using MEGA 11 with the neighbor-joining method supported by a 1,000% bootstrap value showed that all strains belong to *Pseudomonas*. Isolates PsT-04c, PsT-94s, and PsT-116 shared >99% sequence identity with the type strains of *Pseudomonas aeruginosa.* Isolate PsT-124 shared >99% sequence identity with *P. qingdaonensis.* Isolate PsT-130 showed high similarity level to *P. azotoformans* (Figure 8A), and isolate BaT-68s shared >99% sequence identity with *Bacillus paramycoides* (Figure 8B).

## 4 Discussion

Tomato rhizosphere could represent an interesting source of well-adapted PGPR with phosphate solubilizing abilities and biocontrol abilities against phytopathogens. We isolated 100 bacteria from tomato rhizosphere soil using King B medium. King B is a non-selective medium and hence may support the growth of bacteria, belonging to diverse taxa including *Pseudomonas*, *Bacillus,* and *Paenibacillus* (Nam et al., 2016; Bouizgarne et al., 2023; Koo et al., 2023). Furthermore, bacteria were subsequently screened for their ability to solubilize TCP on NBRIP agar medium. Selected highly efficient phosphate solubilizing bacteria (PSB) combined with rock phosphate from Moroccan phosphate mines were used to enhance tomato growth. Among these bacteria, those showing the highest ability to survive inside tomato roots and grow on root exudates were used in order to protect tomato seedlings against *Cmm* by induced systemic resistance. Nautiyal (1999) showed that the NBRIP medium is three times more efficient in terms of demonstrating solubilization compared with Pikovskaya medium (Pikovskaya, 1948). Thus, NBRIP medium used in this investigation was adopted to demonstrate solubilization by the isolates as halos are easy to detect by color change around the colony. In addition, it was demonstrated that solubilization obtained with TCP as sole P source in the medium was significantly greater than in the presence of rock phosphate (Azaroual et al., 2020; Ma et al., 2023). Thus, we used TCP as P source in our experiments. However, qualitative assay on solid medium only allows to detect bacteria, which exhibits solubilization by the production of organic acids. Thus, solubilizing isolates acting by chelating mechanisms could not be detected by this assay. In addition, the importance of the solubilization halo does not necessarily reflect their ability to solubilize in a liquid medium or in the presence of the plant. The decrease in pH during solubilization is probably due to the release of organic acids. The six isolates, PsT-04c, BaT-68s, PsT-94s, PsT-116, PsT-124, and PsT-130, were selected as they showed maximum solubilization ability, in addition to IAA production, and were investigated for the production of organic acids during phosphate solubilization, mainly gluconic acid, maleic acid, and citric acid.

Most microorganisms, particularly *Pseudmonads* and *Bacillus,* set up mechanisms for the solubilization of phosphates based essentially on secreting organic acids and protons (Illmer and Schinner, 1995; Chen et al., 2006; Yahya et al., 2021; Ahmad et al., 2022) or producing phosphatases (Richardson, 2001). During phosphate solubilization, bacteria acidify the periplasmic space by releasing various organic acids of low molecular weight, including lactic acid, gluconic acid, isobutyric acid, acetic acid, glycolic acid, oxalic acid, malonic acid, succinic acid, and 2-ketogluconic acid. Among released organic acids, gluconic acid is the most common in fluorescent *Pseudomonas* spp. (Khan et al., 2009), and it was shown to be involved in the conversion of insoluble phosphate forms into soluble forms, easily assimilated by plants. Consequently, organic acid release leads to an acidification of the external environment of the bacterial cells and a decrease of its pH. As for P solubilization, the major mechanism of K solubilization is conducted by the production of organic and inorganic acids and protons (Meena et al., 2015).

Molecular identification revealed that five strains PsT-04c, PsT-94s, PsT-116, PsT-124, and PsT-130 belong to *Pseudomonas,* while BaT-68s belongs to *Bacillus*. Although PsT-04c, PsT-94s, and PsT-116 are close neighbors of *P. aeruginosa* according to pairwise distance calculated following 16S rRNA gene sequences, they are phenotypically different by their physiological and PGPR traits, suggesting that they represent different strains of the same species. PsT-124 was close to *P. qingdaonesis* and PsT-130 was close to *P. azotoformans,* while BaT-68s was close to *B. paramycoides. Pseudomonas* and *Bacillus* were reported to be powerful phosphate solubilizers (Banerjee et al., 2006; Bouizgarne, 2013; Bouizgarne et al., 2023). *Pseudomonas* are gram-negative bacteria, which inhabit a wide range of environments and exhibit a great metabolic diversity (Stanier et al., 1966). Opposite to *Pseudomonas, Bacillus* are gram-positive spore-forming bacteria that allow them to overcome harsh conditions and survive for extended periods (Bouizgarne, 2013). Both species are well documented as plant growth-promoting rhizobacteria (PGPR). Search for microorganisms with multiple PGPR traits is a promising strategy. Phosphate solubilizing bacteria can promote and improve plant growth parameters (Gamalero et al., 2004; Yahya et al., 2021; Bouizgarne et al., 2023). Beneficial effects of *Pseudomonas* might be due to combined effects of phosphate solubilization, phytohormone production, or biosynthesis of antifungal and antibacterial agents, and these effects usually affects plant size and/or fruit yields positively (Bouizgarne, 2013; Yahya et al., 2021; Bouizgarne et al., 2023). Members of *Bacillus* could trigger plant growth promotion by auxin production or increasing uptake availability of phosphorus (Deepa et al., 2010). The direct effect of *Pseudomonas* spp. and *Bacillus* on plant growth promotion was demonstrated to be due to various mechanisms such as nitrogen fixation, phosphate and iron solubilization, phytohormone modulation, and increased abiotic stress tolerance (Robin et al., 2008; Lugtenberg and Kamilova, 2009; Richardson et al., 2009; Bouizgarne, 2013; Misra and Chauhan, 2020). Thus, six bacterial isolates, which showed maximum solubilization (PsT-04c, BaT-68s, PsT-94s, PsT-116, PsT-124, and PsT-130), were screened in further experiments to test their abilities to trigger other PGPR traits. In addition to phosphate solubilization phosphate, these bacteria showed abilities to solubilize potash and zinc *in vitro*, produce significant amount of the IAA phytohormone, and positively affect the growth of seedlings in the greenhouse when they were applied to tomato. The fact that the selected isolates were able to solubilize phosphate, potash and zinc allow to consider them as potent biofertilizers for tomato. The use of phosphate, potash, and zinc solubilizing bacteria combined with natural phosphate is a promising tool in an eco-friendly strategy, leading to a potential reduction in the application of chemical fertilizers. Different species of *Pseudomonas fluorescens* have been reported as PGPR, and strains belonging to this genus are predominantly found inside the *P. fluorescens* complex (Bouizgarne, 2013; Garrido-Sanz et al., 2016). The application of *Pseudomonas fluorescens* B16 bacteria led to an increase in height, number of flowers, number of fruits, and total fruit weight of tomato plants (Mirza et al., 2001). In addition to phosphate, selected isolates in this investigation showed interesting ability to solubilize insoluble potassium and zinc forms. After nitrogen and phosphorus, potassium is the third important plant nutrient that has a key role in the growth, metabolism, and development of plants. It is involved in increasing plant resistance to biotic and abiotic stresses and is required to activate most different enzymes involved in plant processes (Ahmad et al., 2016). As for phosphorus, potassium is relatively unavailable for plant growth as 90-98% of soil potassium is present as insoluble mineral form (feldspar and mica), and only 2% is available to plants as free soluble form, directly absorbed by plants (Etesami et al., 2017; Jain et al., 2022). Potassium solubilizing bacteria (KSM) can solubilize insoluble potassium minerals such as biotite, feldspar, muscovite, vermiculite, smectite, orthoclase, and mica to soluble forms which could be available to plants (Ahmad et al., 2016; Jain et al., 2022). Species from the genera including *Bacillus*, *Paenibacillus*, *Pseudomonas*, *Burkholderia,* and *Serratia* have been reported to possess the ability of K solubilization (Ahmad et al., 2016; Sun et al., 2020). As for phosphorus, potassium is a macronutrient whose concentration in the soil might limit plant growth (Parmar and Sindhu, 2013), and our isolates could probably also enhance its solubilization or uptake from soil. Zinc is among the essential micronutrients for plant growth, and inorganic zinc in soil is unavailable form for plant assimilation. Thus, the selected bacteria could be involved in making the insoluble inorganic zinc into biologically available form for plants. Zinc plays an essential role in the biosynthesis of IAA through the formation of its precursor, tryptophan. In addition, zinc is involved in many other physiological functions: cell division, elongation, fruit development, and control of gene expression, stabilizing RNA and DNA structure and maintaining the activity of RNA-degrading enzymes (Brown et al., 1993). Although not measured in Bunt and Rovira liquid medium in this investigation like for NBRIP, it is suggested that zinc solubilization could be due to a drop in pH and organic acid production, potentially involved in K solubilization (Fasim et al., 2002; Ahmad et al., 2016). Moreover, as for phosphorus, it has been reported that inoculation with potassium and zinc solubilizing-bacteria led to beneficial effects on the growth of different plants (Bakhshandeh et al., 2017; Ali et al., 2020; Batool et al., 2021). In addition, indole-3-acetic acid is the most common auxin phytohormone, involved in cell division, root elongation, fruit development, and senescence, and it is the most important auxin produced by bacteria, plants, and fungi (Bouizgarne, 2013; Lesueur et al., 2016). Its role in the observed positive beneficial effects of the six isolates is probably crucial. Chu et al. (2020), reported positive effects of *Pseudomonas* PS01 on development of the root system by triggering lateral root and root hair formation, due to the production of IAA by the bacterium. All those potential beneficial effects demonstrated *in vitro*, make them good candidates for application as inoculants for tomato growth promotion. In this investigation, treatment by the six bacteria led to an improvement in growth parameters: shoot and root length of seedlings and their fresh and dry weight.

Photosynthesis plays a key role in metabolic processes by which plants grow. The selected bacteria increased the chlorophyll content in plant leave tissues, which could be involved in enhanced photosynthesis. In this investigation, some isolates showed a positive effect on chlorophyll a and b and total chlorophyll. Research studies, aiming to test the effectiveness of PGPR on nutrient parameters, demonstrated that their application resulted in a significant increased total chlorophyll, sugar, and protein contents (Pirlak and Köse, 2009; Khan et al., 2019). On the other hand, the absence of any changes in chlorophyll fluorescence after 60 days of culture suggests that our isolates did not induce any biotic stress to plants. The inoculation by selected bacterial isolates resulted in positive effects on the mineral element contents of tomato seedlings after 60 days. Indeed, following application of the bacterial strains, an increase in leaves P content and K, Na and Ca contents in shoot was recorded for all the isolates. Enhancement in exchangeable cations was shown in many plants, following treatments with PGPR bacteria. Increased mineral nutrition, improved water and nutrient translocation, efficient nutrient uptake and utilization, increased photosynthesis, and the ability to enhance N, Ca, Mg, and K uptake by plants are important beneficial effects of the plant growth-promoting rhizobacteria (Abd-Allah et al., 2018; Kour et al., 2020). A study by Ipek et al. (2014) demonstrated an increase in plant tissue nutrients, such as nitrogen, phosphorus, potassium, calcium, iron, copper, manganese, and boron by PGPR treatments. Those effects could be displayed even under harsh environmental conditions (Jha and Subramanian, 2018; Khan et al., 2019). High total sugars and proteins contents shown in treated plants with the phosphate solubilizing bacteria, compared to control, may be due to increased leaf biomass, to increased availability of assimilates due to increased photosynthesis and availability of nutrients to the plants following the application of plant growth promoting bacteria.

Due to their various antagonistic properties against phytopathogens, fluorescent *Pseudomonas* and *Bacillus* are the most studied bacteria regarding their usefulness as biocontrol agents of phytopathogens (Lemanceau et al., 2007; Bouizgarne, 2013; Bouizgarne and Ouhdouch, 2017). Another indirect mechanism is known as induced systemic resistance (ISR) which allows plants to protect themselves against fungal and bacterial disease agents (Chen et al., 2020; Yahya et al., 2021; Abdelaziz et al., 2023; Bellotti et al., 2023). Bacteria belonging to *Pseudomonas* and *Bacillus* strains were described as resistance inducers in tomato phytopathogens (Gautam et al., 2019; Bellotti et al., 2023). Thus, the selection of bacterial isolates which could induce resistance could be adopted as a strategy for biocontrol of bacterial canker. In this study, we selected two *Pseudomonas* isolates PsT-04c and PsT-130 as ISR inducers against an aggressive isolate of *Cmm.* The bacteria selected in this study showed ability to grow on root tissues and root exudates. Root exudates are carbon-rich rhizodeposits containing primary and secondary low molecular weight metabolites containing multiple carbohydrates (amino acids, organic acids, and soluble phenolic compounds), which are secreted by plant roots. The role of the root exudates in plant–microbe interactions and plant responses to various phytopathogens is well established (Kandel et al., 2017; Mavrodi et al., 2021; Yahya et al., 2021). It was demonstrated that root exudates initiate the communication between soil bacteria and host plants, leading to bacterial adhesion to the root surfaces. Root exudates also modulate rhizosphere microbial regulation of phosphorus uptake (Sasse et al., 2018) and stimulate beneficial microbial agents including biocontrol agents of plant pathogens (Zhang et al., 2014; Wang et al., 2019; Wen et al., 2021). Thus, we selected the best growing isolates on root tissues and root exudates, PsT-04c and PsT-130 for root treatments in greenhouse resistance induction trials. Treatments with these bacteria provided protection against bacterial canker disease and increased H_2_O_2_ levels. Treatments allowed a significant increase in the levels of root soluble phenolic compounds, for which the role as defense molecules of plants against phytopathogens is well established. An increase was also noticed in phenylalanine ammonia-lyase (PAL) activity, explaining the increase in soluble phenolic compounds. Indeed, PAL is the key enzyme involved in the phenylpropanoid pathway of the phenolic compound synthesis. The role of PAL in tomato resistance to bacterial canker was reported by Umesha (2006). The results of protection of tomato seedlings against the causative agent of bacterial canker can be explained by the marked increase in the content of soluble phenolic compounds and hydrogen peroxide (H_2_O_2_). While infection of the plant with *Cmm* did not significantly increase the H_2_O_2_ level (0.125 μg g^−1^ FW) compared with the control seedlings (0.115 μg g^−1^ FW), this level was almost doubled during the PsT-04c and PsT-130 treatments with approximately 0.200 μg g^−1^ FW. Accumulation of H_2_O_2_ was reported as the mechanism of induced systemic resistance, following treatment by *Pseudomonas* biocontrol agents (De Vleesschauwer and Hoefte, 2006; Bellameche et al., 2021). H_2_O_2_ plays a central role in plant resistance to pathogens as it can activate several defense reactions including the strengthening of physical barriers and development of the hypersensitivity reaction. Phenolic compounds are secondary metabolites that are commonly present in plant tissues and are known for their intervention in the defense of plants against phytopathogens. We suggest that these bacteria act by inducing systemic resistance in tomatoes, allowing protection against the pathogen. The involvement of other enzymes, such as catalase, peroxidase, polyphenol oxidase, or superoxide dismutase (Yahya et al., 2021), is not excluded and still to be investigated. The induction of systemic resistance (ISR) by the two *Pseudomonas* bacteria is an excellent way to boost the defense of the tomato against a post challenge with *Cmm*. On the other hand, those two isolates showed antagonistic activity against *Cmm* growth *in vitro* (unpublished data) by the production of antibiotics (Raaijmakers et al., 2002), and it is not excluded that this activity could also contribute to the protective effect against *Cmm* at ground level, which is found in our experience.

In conclusion, isolated fluorescent pseudomonad and *Bacillus* sp. have PGPR effect that improves growth parameters. In this study, we isolate phosphate, potash, and zinc-solubilizing bacteria including five fluorescent pseudomonads and one *Bacillus*. The application of these bacteria in combination with Moroccan rock phosphate led to an increase in tomato shoot and root length and their fresh and dry weights. Phosphate solubilizing bacteria showing beneficial effects on tomato growth parameters showed abilities to improve to different extents, the measured physiological parameters. The triggering of defense mechanisms of tomato by two *Pseudomonas* strains suggests that they are interesting as bio primers for biocontrol of bacterial canker disease. Research on these two areas will help widen the alternative options available for tomato crop fertilization and managing bacterial canker disease. Experiments, aiming to use the two inducing bacteria and other *Pseudomonas* and *Serratia* strains, showing *in vitro* antibiosis against *Cmm* (Bouizgarne et al., 2023; Bouizgarne and Bakki, 2024), are in progress, as well as bioformulations of most efficient bacteria in combination with rock phosphate are applied as biofertilizers and biopesticides.

## Data availability statement

The datasets presented in this study can be found in online repositories. The names of the repository/repositories and accession number(s) can be found at: https://www.ncbi.nlm.nih.gov/genbank/, OP677775, OP677776, OP677777, OP677861, OP677782, and OP677783.

## Author contributions

MB: Conceptualization, Investigation, Methodology, Visualization, Software, Data curation, Writing – original draft. BaB: Formal analysis, Software, Visualization, Investigation, Methodology, Writing – review & editing. OM: Investigation, Methodology, Writing – review & editing. QE: Writing – review & editing, Software. AH: Resources, Writing – review & editing. EB: Writing – review & editing. KA: Resources, Writing – review & editing. BrB: Conceptualization, Data curation, Funding acquisition, Investigation, Methodology, Project administration, Resources, Software, Supervision, Validation, Visualization, Writing – original draft, Writing – review & editing.
