# Supplementary material for: Phosphate solubilizing Pseudomonas and Bacillus combined with rock phosphates promoting tomato growth and reducing bacterial canker disease
Source: Front Microbiol. 2024 May 3;15:1289466. doi: 10.3389/fmicb.2024.1289466 (PMC11100333; doi:10.3389/fmicb.2024.1289466)
Supplement: Supplementary file 1 [file Image_1.pdf]

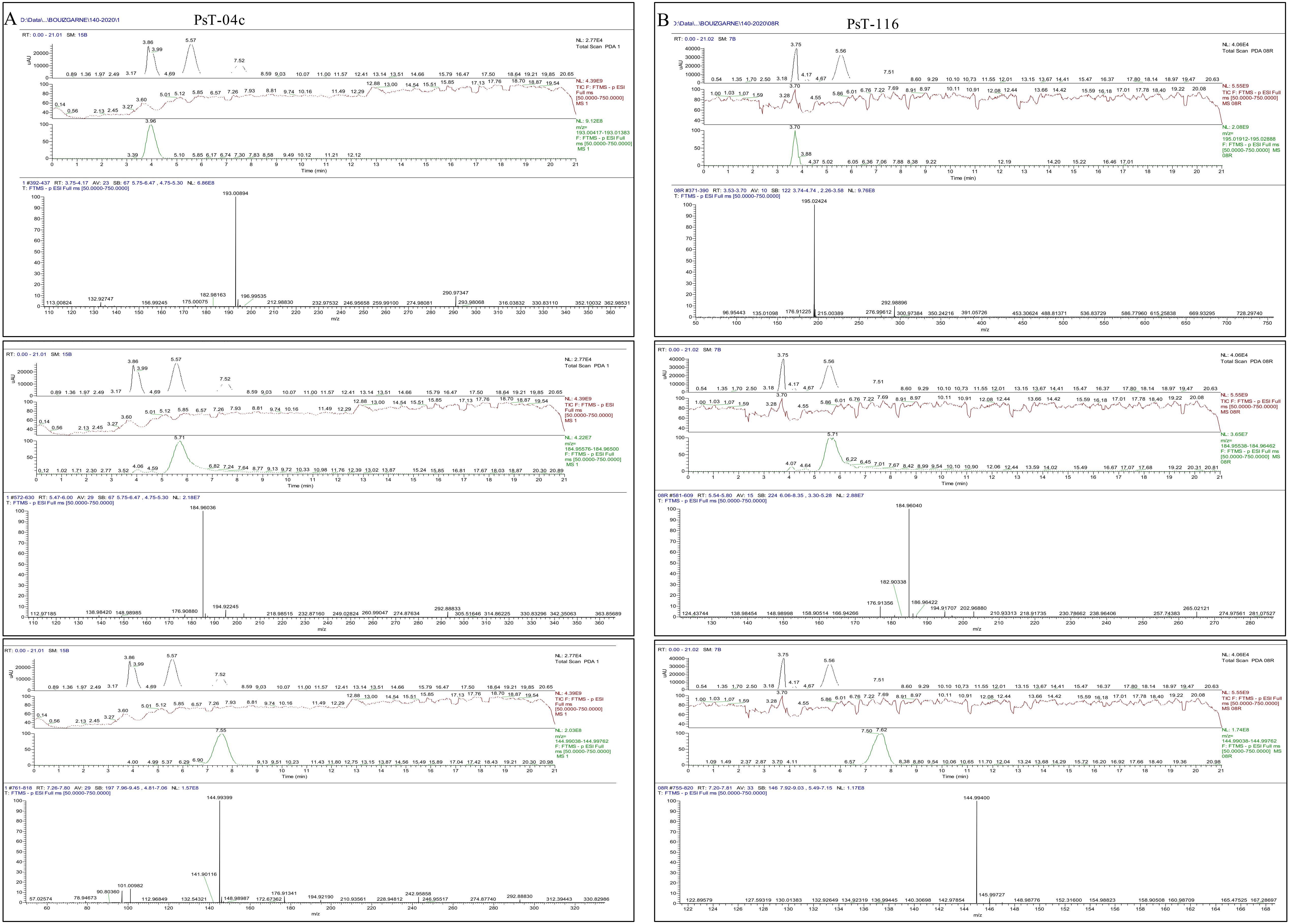

Supplementary Figure : MS spectra of the filtered supernatant from NBRIP liquid cultures of isolates PsT-04c and PsT-130
